# Analysis of Brugada syndrome loci reveals that fine-mapping clustered GWAS hits enhances the annotation of disease-relevant variants

**DOI:** 10.1016/j.xcrm.2021.100250

**Published:** 2021-04-20

**Authors:** Mel·lina Pinsach-Abuin, Bernat del Olmo, Adrian Pérez-Agustin, Jesus Mates, Catarina Allegue, Anna Iglesias, Qi Ma, Daria Merkurjev, Sergiy Konovalov, Jing Zhang, Farah Sheikh, Amalio Telenti, Josep Brugada, Ramon Brugada, Melissa Gymrek, Julia di Iulio, Ivan Garcia-Bassets, Sara Pagans

**Affiliations:** 1 Department of Medical Sciences, School of Medicine, Universitat de Girona, Girona, Spain; 2 Visiting Scholar Program, Department of Medicine, School of Medicine, University of California, San Diego, La Jolla, CA, USA; 3 Institut d’Investigació Biomèdica de Girona, Salt, Spain; 4 Centro de Investigación Biomédica en Red de Enfermedades Cardiovasculares, Madrid, Spain; 5 Department of Medicine, School of Medicine, University of California, San Diego, La Jolla, CA, USA; 6 Department of Statistics, University of California, Los Angeles, Los Angeles, CA, USA; 7 Department of Integrative Structural and Computational Biology, The Scripps Research Institute, La Jolla, CA, USA; 8 Arrhythmia Unit, Hospital Clinic de Barcelona, Universitat de Barcelona, Barcelona, Spain; 9 Cardiology Service, Hospital Universitari Dr. Josep Trueta, Girona, Spain; 10 Department of Computer Science and Engineering, University of California, San Diego, La Jolla, CA, USA

**Keywords:** GWAS, haplotype block, common SNV, non-coding SNV, SNV redundancy, risk haplotype, protective haplotype, long-read sequencing, sudden cardiac death, Brugada syndrome

## Abstract

Genome-wide association studies (GWASs) are instrumental in identifying loci harboring common single-nucleotide variants (SNVs) that affect human traits and diseases. GWAS hits emerge in clusters, but the focus is often on the most significant hit in each trait- or disease-associated locus. The remaining hits represent SNVs in linkage disequilibrium (LD) and are considered redundant and thus frequently marginally reported or exploited. Here, we interrogate the value of integrating the full set of GWAS hits in a locus repeatedly associated with cardiac conduction traits and arrhythmia, *SCN5A*-*SCN10A*. Our analysis reveals 5 common 7-SNV haplotypes (Hap1–5) with 2 combinations associated with life-threatening arrhythmia—Brugada syndrome (the risk Hap^1/1^ and protective Hap^2/3^ genotypes). Hap1 and Hap2 share 3 SNVs; thus, this analysis suggests that assuming redundancy among clustered GWAS hits can lead to confounding disease-risk associations and supports the need to deconstruct GWAS data in the context of haplotype composition.

## Highlights

Haplotype block analysis separates *cis*-regulatory regions around *SCN5A* and *SCN10A*
Haplotype diversity limits to 5 common 7-SNV combinations in the enhancer region
Hap^1/1^ and Hap^2/3^ are Brugada syndrome risk and protective genotypes, respectively
Hap^1/1^ and Hap^2/3^ genotypes are anti-correlated with *SCN10A* expression

## Graphical abstract

**Figure:**
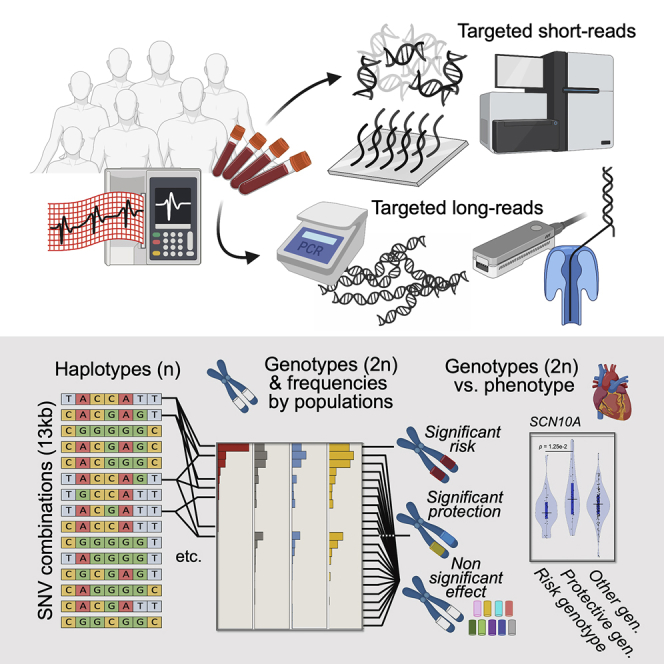

## Teaser

The GWAS field tends to focus on the most significant hit in each disease-associated locus, despite that its distinct association is not a proxy of functionality and it is accompanied by other significant clustered signals. Focusing on Brugada syndrome, Pinsach-Abuin et al. show that deconstructing GWAS hit clusters enhances disease-relevant annotations.

## Introduction

For over a decade, genome-wide association studies (GWASs) have laid the foundation for identifying heritable traits and disease-associated features.^1^ GWASs compare common single-nucleotide variants (SNVs) between populations differing in a phenotypic trait—often a clinical symptom—to identify trait/disease-associated variants. In each trait/disease-associated locus, the most significant associations (GWAS hits) emerge in clusters, with a lead SNV (the variant with the highest significance) surrounded by SNVs with lower but still significant association signals. However, the strength of the individual associations is not a readout of functionality and can vary across populations.^2^ Despite this fact, the GWAS field has historically focused on the lead SNVs and has inferred redundancy on the remaining associations, often reported in supplemental information for this reason. The observation of hits in clusters fits with a model of “haplotype blocks,” or genomic regions inherited as single sets (called haplotypes) across generations with internal SNVs transmitted in linkage disequilibrium (LD).^3^, ^4^, ^5^, ^6^, ^7^, ^8^, ^9^, ^10^

Fine-mapping SNVs or accounting for SNV multiplicity (rather than for single variants in a locus) improves GWAS accuracy.^11^, ^12^, ^13^, ^14^ Thus, we postulated that integrating the full set of GWAS hits in a cluster may enhance the information generated by GWAS data. To test this analytical decision, we focused on Brugada syndrome, a rare condition responsible for 12% of sudden cardiac deaths.^15^^,^^16^ Brugada syndrome is a cardiac electrical disorder characterized by ventricular arrhythmias leading to a high risk of sudden cardiac arrest.^17^ Notably, most cases with a known genetic feature (75%) carry a rare deleterious variant in the *SCN5A* gene.^18^, ^19^, ^20^ Among the rest, some carry a rare deleterious variant in the *SCN10A* gene.^21^, ^22^, ^23^ These two genes are separated by 50 kb along chromosome 3 and encode the pore-forming α-subunits of the voltage-gated sodium channels Na_V_1.5 and Na_V_1.8, respectively, which are critical to propagate action potentials.^24^

Despite the overwhelming consistency in detecting rare deleterious variants, particularly in the *SCN5A* gene, 65% of the Brugada syndrome cases cannot be explained by a genetic feature.^25^ However, as the etiology of this condition is attributed mainly to a genetic origin, it is possible that the *SCN5A-SCN10A* locus carries missing Brugada heritability in the form of other genetic features. In line with this hypothesis, the *SCN5A-SCN10A* locus also harbors common trait/disease-associated SNVs.^1^ These variants have been found in exonic, intronic, and untranslated (UTR) regions of the *SCN5A* gene,^26^, ^27^, ^28^, ^29^, ^30^ the *SCN5A* promoter,^31^, ^32^, ^33^ and around an enhancer located in an intronic region of the *SCN10A* gene.^34^, ^35^, ^36^ At the *SCN5A* promoter, for example, a haplotype of six common SNVs (called HapB) is associated with low *SCN5A* promoter activity and an altered electrocardiogram (ECG) in individuals of Asian descent.^31^ An intronic enhancer in the *SCN10A* gene carries a cluster of GWAS hits associated with Brugada syndrome.^34^ Some of the underlying common SNVs, other than the lead, have also been proposed to modulate *SCN5A* expression.^34^^,^^35^ Still, it remains unclear whether these SNVs and the lead SNV belong to the same haplotype associated with this heart condition. Here, we characterize the haplotype block structure in the *SCN5A-SCN10A* locus and annotate the block containing the most clustered GWAS hits to interrogate the value of deconstructing disease-associated SNV clusters and tackle the heritability gap in Brugada syndrome.

## Results

### Analysis of haplotype block structure in the *SCN5A-SCN10A* locus

**Figure 1:**
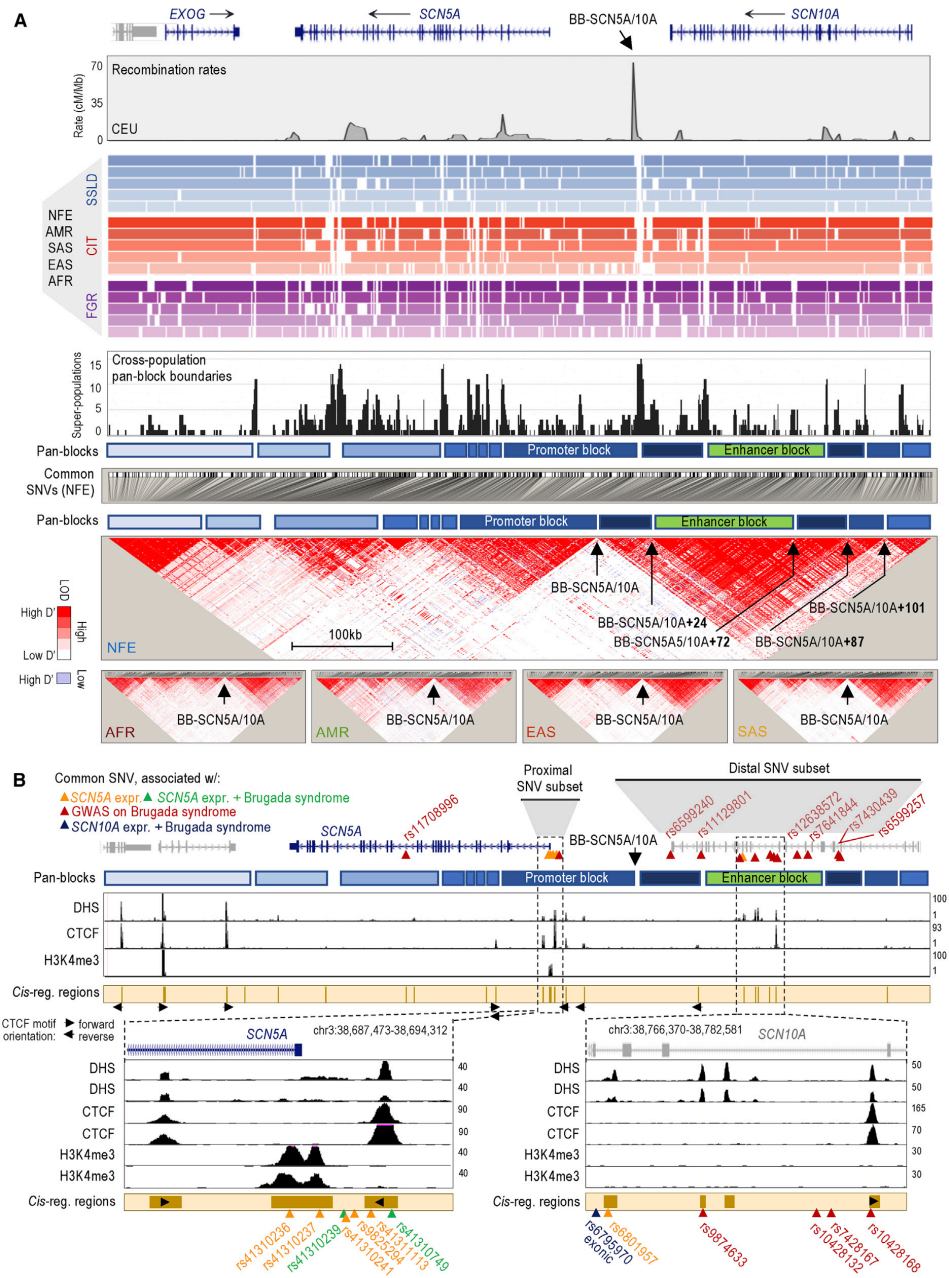
Haplotype block frameworks in the *SCN5A-SCN10A* locus (A) The top profile shows HapMap rate estimates of recombination in the CEU population in cM (centimorgans)/Mb (megabase). Genomic coordinates (hg19): chr3:38,516,506-38,841,720. The colored tracks depict haplotype-block estimates by super-population (from top to bottom: NFE, AFR, AMR, EAS, and SAS) using 3 block-partitioning methods (SSLD, CIT, and FGR) implemented in Haploview version 4.2. A summary profile of the predicted pan-blocks/pan-boundaries (in black) shows the sum of the times a block boundary is imputed in a population with a given method (max value = 15 for 5 populations with three methods). Note: <100-bp boundaries may not be visible in the colored tracks, yet will be accounted for in the summary profile. Tracks in blue represent the approximated locations of the predicted pan-blocks/pan-boundaries (top track: relative to genomic coordinate; bottom track: relative to SNV). The large heatmap represents a LD plot of n = 697 SNVs with MAF ≥5% in NFE, and the spatial distribution of LD coefficient (D’) values and the likelihood of odds (LOD) ratios for each pairwise SNV set based on the 1KG Phase 3 dataset. The rest of LD plots correspond to n = 967 AFR, n = 838 AMR, n = 728 EAS, and n = 736 SAS. Predicted pan-boundaries in the NFE plot are indicated: BB-SCN5A/10A (chr3:38,724,850-38,727,325), BB-SCN5A/10A+24 (chr3:38,751,191-38,752,018), BB-SCN5A/10A+72 (chr3:38,798,839-38,799,304), BB-SCN5A/10A+87 (chr3:38,814,174-38,814,220), and BB-SCN5A/10A+101 (chr3:38,828,333-38,829,269). Only the BB-SCN5A/10A site is indicated in the rest of the LD plots. See also Data S1, Table S1. (B) Genomic location of relevant common SNVs and maps of DHS, CTCF occupation, and H3K4me3 accumulation in human cardiac myocytes (data sources: GSM736516/GSM736504, GSM1022657/GSM1022677, and GSM945308). The cantaloupe-colored track depicts the sum of chromatin features. CTCF motifs and orientation also indicated. Genomic coordinates based on hg19. See also Figure S1.

Rate estimates of recombination can be used to broadly infer haplotype block structure.^3^^,^^7^^,^^37^ As previously shown,^34^^,^^35^ HapMap-generated profiles of recombination estimates suggest multiple hotspots across the *SCN5A-SCN10A* locus (depicted as peaks in Figure 1A, top panel).^38^ These sites represent major candidate haplotype block boundaries (in the CEU population, Utah residents of northern and western European descent in Figure 1A, top panel). The most consistent boundary resides 34 kb upstream of the *SCN5A* gene and 14 kb downstream of the *SCN10A* gene, henceforth called block boundary BB-SCN5A/10A (Figure 1A). This boundary partitions 2 large sections of LD in the 5 human super-populations of the 1000 Genomes (1KG) Project: non-Finnish Europeans (NFE), Africans (AFR), Americans (AMR), South East Asians (SAS), and East Asians (EAS)^38^^,^^39^ (Figure 1A, bottom, LD heatmaps). Three block-partitioning methods—solid spine of LD (SSLD), confidence interval test (CIT), and 4-gamete rule (FGR)—infer this site across the 5 human super-populations (Figure 1A, colored tracks; Data S1, Table S1). These three methods also provide a high-resolution map of haplotype-block structure, which allowed us to predict additional haplotype block boundaries (Figure 1A, colored tracks). However, as each block-partitioning method relies on different LD principles,^40^ some of these predictions were discordant. We annotated the most consistent predictions across methods and super-populations, referring to them as “pan-block” boundaries (the highest peaks in the black profile shown in Figure 1A). We also annotated the regions between pan-block boundaries as pan-blocks (Figure 1A, track). Interestingly, we observed that only 2 pan-blocks accumulate GWAS hits associated with Brugada syndrome and common SNVs associated with *SCN5A* or *SCN10A* expression (Figure 1B, proximal and distal SNV subsets).^19^^,^^31^^,^^32^^,^^34^^,^^41^ In particular, one pan-block, 48 kb long, resides immediately downstream of BB-SCN5A/10A (chr3:38,671,769-38,724,849), and the second pan-block, 52 kb long, resides between 24 and 72 kb upstream of BB-SCN5A/10A (“BB-SCN5A/10A+24kb” and “BB-SCN5A/10A+72kb,” respectively; Figures 1A and 1B). The sizes of these 2 pan-blocks are in the range of the average block length in the European population, 34.8–54.4 kb.^42^ Moreover, ENCODE-generated maps of chromatin features in tissue-isolated cardiac myocytes (DNase I hypersensitive sites [DHS]; CTCF occupation; and H3K4me3 accumulation) confirm that these 2 pan-blocks contain the *SCN5A* promoter and an *SCN10A*-intronic enhancer (Figure 1B; DHS, CTCF, and H3K4me3 tracks).^43^, ^44^, ^45^

### The enhancer pan-block accumulates a wide amplitude of Brugada association signals

In a previous GWAS on Brugada syndrome, Bezzina and colleagues^34^ reported a cluster of hits with a lead SNV, rs10428132, surrounded by 9 other significant association signals across the intronic regions of the *SCN10A* gene (Figure 1B; distal SNV subset, GWAS SNVs). In the same study, a confirmatory analysis revealed an additional lead association at an intronic region of the *SCN5A* gene (Figure 1B; rs11708996).^34^ According to our estimates, the largest subset of these 11 GWAS hits (n = 6) resides in the enhancer pan-block (Figures 1B and S1; GWAS hits, array: rs9874633, rs10428132, rs7428167, rs10428168, rs12638572, and rs7641844). However, the hits exhibit a wide amplitude of association signals, ranging from p = 6.79e−26 to p = 3.80e−08.^34^ We aimed to investigate how this wide dispersion of signals, while typical in clustered GWAS data, could be reconciled with a model of consistent SNV co-transmission within the pan-block.^46^ We also aimed to interrogate how this wide dispersion of signals could be reconciled with the model of SNV redundancy.^46^

**Figure 2:**
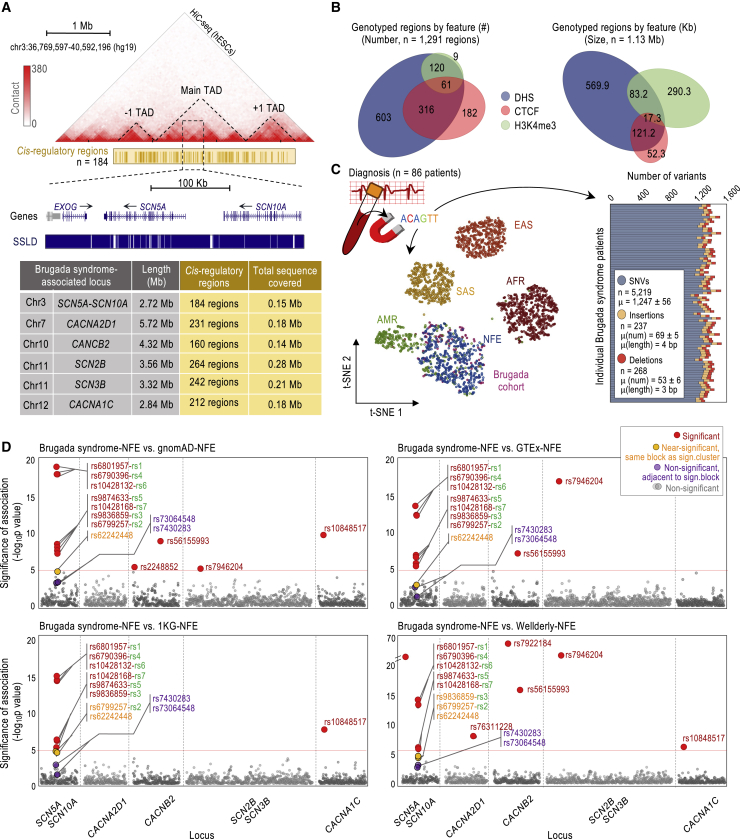
Deep genotyping of *cis*-regulatory regions in Brugada-associated loci (A) Heatmap of long-range chromatin interactions to delineate TAD structure (Hi-C-seq; data source: GSM862723/GSM892306). TAD boundaries set according to Dixon et al.^47^ Cantaloupe-colored track depicts DHS/CTCF/H3K4me3 regions across 3 TADs in human cardiac myocytes (data sources: GSM736516/GSM736504, GSM1022657/GSM1022677, and GSM945308). Blue tracks depict SSLD-based haplotype-block estimations. Table shows the size (Mb) of the 3 TADs selected for each Brugada syndrome-associated locus, the number of *cis*-regulatory regions, and their total size (Mb). (B) Summary of *cis*-regulatory regions by chromatin feature (by class or base pairs). The 2 manually included regions are represented only on the right panel. See also Data S1, Table S2. (C) Ancestry and variant annotation based on genotyping of *cis*-regulatory regions captured from blood-derived genomic DNA of n = 86 Brugada syndrome cases (type I ECG-based diagnosis). The t-SNE plot, based on the first 6 principal components, shows the ancestry admixture for the Brugada syndrome cases in the context of the 1KG Phase 3 human super-populations. Graph shows the summary of SNVs/insertions/deletions in the Brugada cohort (total number, *n*; mean number, μ [number]; and mean size μ [length] for indels). See also Figure S2 and Data S1, Table S3. (D) Manhattan plots showing the significance of the associations for n = 2,121 common SNVs in the Brugada cohort in a case-control analysis of n = 86 Brugada syndrome cases using n = 7,718 NFE individuals (gnomAD) as controls (top left), n = 355 (GTEx; top right), n = 404 (1KG Phase 3; bottom left), and n = 196 (Wellderly; bottom right) NFE individuals. Relevant SNVs labeled as indicated. Significance tested by Fisher’s exact test. The red line marks the threshold of significance (Bonferroni-corrected α level of p value 2.36e−5 [0.05/2,121] according to the number of common SNVs tested). See also Figure S2 and Data S1, Table S4.

Of note, the mentioned GWAS on Brugada syndrome was based on the Axiom Affymetrix platform and focused on SNVs with minor allele frequencies (MAFs) equal or higher than 10%.^34^ For our analyses, in contrast, we used targeted next-generation sequencing (NGS) technology, which allowed us high-depth genotyping across selected regions at the enhancer-containing pan-block. Moreover, we were interested in a larger subset of SNVs, in particular, those with MAFs equal or higher than 0.5%, which covers common (MAF ≥ 5%) and low-frequency (MAF = 0.5%–5%) variants.^39^ For practicality, we focused on SNVs that may have a *cis*-regulatory role in transcription in cardiac myocytes, as these variants would be more likely to be functionally relevant. To identify the regions of interest, we annotated DHS, as proxies of transcription factor occupation, in the topologically associated domain (TAD) of the *SCN5A-SCN10A* locus and the upstream and downstream TADs, which ensured that a large set of potential *cis*-regulatory regions at and around this locus were covered (Figure 2A; long-range interactions heatmap, TAD; see STAR Methods for more details).^44^^,^^47^ In addition, we annotated H3K4me3-enriched and CTCF-occupied regions in the 3 TADs,^44^^,^^45^ as these regions are known to carry high SNV densities (Figure 2A; see also Figure 1B, bottom panels, for a magnification, *cis*-regulatory regions track).^48^

Cross-referencing the annotated regions with prior Brugada GWAS data, we found that only 2 of the 6 GWAS hits in the enhancer-containing pan-block (rs9874633 and rs10428168) and 3 (out of 6) SNVs with a proposed modulatory role in *SCN5A* transcription on the promoter-containing pan-block (rs41310236, rs41310237, and rs41311113) were covered by our annotations (Figure 1B).^19^^,^^31^^,^^32^^,^^34^^,^^41^ For this reason, we also added to our annotations the regions containing the 2 lead SNVs that were previously associated with Brugada syndrome (rs10428132 and the isolated rs11708996), which do not apparently overlap with DHS, H3K4me3, or CTCF binding (Figures 1B and S1; NGS-based genotyping). Importantly, the annotated regions in the enhancer-containing pan-block included 7 additional common SNVs that did not emerge as hits in the prior GWAS; yet, it remains unclear whether these SNVs were examined (rs6801957, rs6799257, rs9836859, rs6790396, rs62242446, rs62242447, and rs62242448; Figure S1; NGS-based genotyping). In total, the annotated regions contained 10 common SNVs in the enhancer-containing pan-block and many others in the surrounding pan-blocks and across the locus, which are sufficient for our purposes. To maximize our sequencing efforts, we also annotated DHS, H3K4me3, and CTCF-occupied regions at and around 5 other Brugada-associated loci: *SCN2B, SCN3B, CACNA1C, CACNA2D1*, and *CACNB2* (Figure 2A).^49^, ^50^, ^51^, ^52^ In total, the regions for genotyping were n = 1,291 *cis*-regulatory regions annotated using chromatin features and n = 2 non-*cis*-regulatory regions annotated manually, which together represent 1.13 Mb of genomic DNA (Figures 2A and 2B; Data S1, Table S2).

Using the Illumina Nextera system, we captured the n = 1,293 regions from blood-extracted genomic DNA of n = 86 unrelated Brugada syndrome cases. These cases were diagnosed with a type I ECG pattern, characterized by a “coved” ST-segment elevation on the right precordial leads detected either at baseline or after challenge using sodium channel blockers (ajmaline or flecainide) typically used to unmask elusive Brugada arrhythmias.^17^ Most cases were males in their 40s and asymptomatic, common in Brugada syndrome (Table 1). We note that we pre-selected those cases that do not carry deleterious variants in *SCN5A* coding regions with the purpose of enriching for Brugada syndrome cases with currently unknown Brugada-associated genetic features (see STAR Methods). After sequencing, we obtained n = 3.8 ± 0.95 million on-target reads for each patient (target enrichment of 64.79%), with an average coverage of n = 384x ± 149x. Using GATK HaplotypeCaller^53^ and stringent filtering conditions (see STAR Methods), we annotated n = 1,247 ± 56 SNVs, n = 69 ± 5 insertions, and n = 53 ± 6 deletions for patient (Figure 2C, graph). We used this SNV panel to confirm the NFE descent of the n = 86 Brugada syndrome cases (Figure 2C, t-distributed stochastic neighbor embedding [t-SNE] plot).

**Table 1:** Clinical characteristics of the Brugada syndrome cases included in the study

	Males	Females	Total
No. cases (%)	67 (77.9)	19 (22.1)	86 (100)
Age at diagnosis, y^a^	47 (±12)	49 (±12)	47 (±12)
Spontaneous type 1 ECG pattern (%)	27 (40.3)	8 (42.1)	35 (40.7)
Type 1 ECG pattern induced by sodium channel blocker (%)	40 (59.7)	11 (57.9)	51 (59.3)
Symptomatic, resuscitated cardiac arrest and/or syncope (%)	13 (19.4)	5 (26.3)	18 (20.9)
Family history of sudden death (%)	21 (31.3)	5 (26.3)	26 (30.2)
ICD (%)	27 (40.3)	5 (26.3)	32 (37.2)

ECG, electrocardiogram. ICD, implantable cardioverter defibrillator.

a Results presented as averages ± SDs.

To identify SNVs with MAF ≥0.5% in individuals of NFE descent, we next cross-referenced the SNVs identified with variants from the Genome Aggregation Database (gnomAD) for NFE individuals.^54^ We annotated n = 1,232 low-frequency SNVs (MAF = 0.5%–5%) and n = 2,121 common SNVs (MAF ≥ 5%) and conducted a case-control association analysis using n = 7,718 NFE individuals from the gnomAD database as controls.^54^ We note that this control group was not subject to ECGs for Brugada syndrome diagnosis, but it is not expected to have as high of a prevalence of Brugada syndrome cases as the case cohort, for which we refer to it as “control”. After applying the Bonferroni correction, we identified n = 11 significantly enriched SNVs (Figure 2D, top left panel; Figure S2A; Data S1, Table S4, gnomAD-NFE columns). As expected, the most significant associations reside in the enhancer-containing pan-block, and fitting with a model of haplotype blocks, most (7 of 10) show a significant association to Brugada syndrome (Figures 2D and S1). This group includes the 3 Brugada GWAS hits covered in our analysis in the enhancer-containing pan-block and 4 novel variants (Figure S1). One of the 4 novel SNVs (Figure 2D, top left panel, in red) corresponds to the lead SNV in our analysis, rs6801957 (hereafter referred to as rs1). The neighboring SNVs correspond to rs6799257 (rs2, novel), rs9836859 (rs3, novel), rs6790396 (rs4, novel), rs9874633 (rs5, GWAS hit), rs10428132 (rs6, the lead GWAS hit), and rs10428168 (rs7, GWAS hit; Figures 2D and S1). An eighth SNV (out of the 10), rs62242448, reached sub-threshold significance (p = 3.34e−5; Figure 2D, top left panel, in orange), and only 2 SNVs (low-frequency cases, 2 out of the 10) did not reach significance or sub-threshold significance (rs62242446, p = 1.000, and rs62242447, p = 0.857; Figures S1 and S2A). As in GWASs, we observed a wide dispersion of association signals, ranging from p = 2.30e−19 to p = 3.83e−8—wider if we consider the 3 non-significant SNVs. In the case of low-frequency variants (MAF = 0.5%–5%), we did not detect any other association in the enhancer-containing pan-block (Figure S2A; Data S1, Table S4, gnomAD-NFE columns).

Before moving forward to interrogate the basis of the wide amplitude in association signals within the enhancer-containing pan-block, we sought to first replicate the 4 novel Brugada-associated SNVs (rs1–4) using additional independent controls of ancestry-matched individuals (NFE). In particular, we compared the Brugada cohort to n = 404 of the 1KG Project Phase 3,^39^ n = 196 of a healthy aging cohort known as Wellderly,^55^ and n = 355 of the Genotype-Tissue Expression (GTEx) dataset^56^ (Figures 2D and S2B). Using the GTEx-NFE dataset, we replicated the 7 significant association signals, rs1–7, including the 4 novel SNVs (Figure 2D, top right panel; Data S1, Table S4, GTEx-NFE columns). Using the 1KG-NFE dataset, we replicated 3 of the 4 novel association signals (rs1/3/4) and rs5–7, but not rs2 (Figure 2D, bottom left panel; Data S1, Table S4, 1KG-NFE columns). Using the Wellderly-NFE dataset, we replicated 2 of the 4 novel association signals (rs1/4) and rs5–7, but not rs2/3 (Figure 2D, bottom right panel; Data S1, Table S4, Wellderly-NFE columns). In the two instances in which rs2/3 were not replicated, however, these SNVs reached sub-threshold significance (orange labels in Figure 2D; Data S1, Table S4), which may still represent real associations as they overlap with chromatin features.^57^ The Bonferroni method is also notorious for being overly conservative and prone to false negatives with small sample sizes, which may also suggest that these 2 sub-threshold association signals could be, in fact, real.^58^^,^^59^

### The most common haplotypes in the pan-block are associated with Brugada syndrome

**Figure 3:**
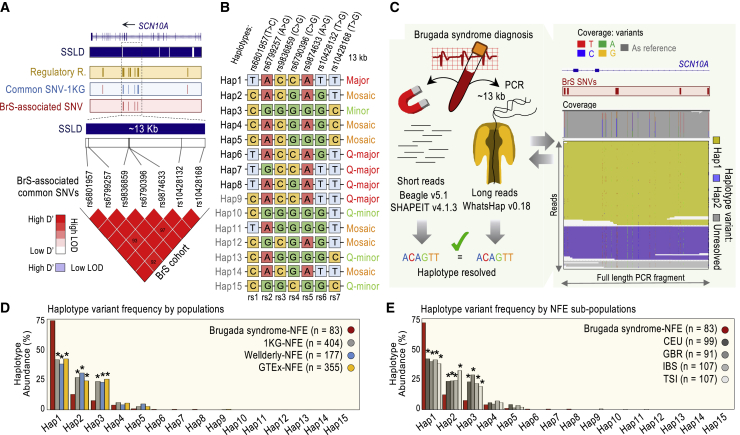
A catalog of haplotypes in the enhancer pan-block (A) Seven common SNVs (according to gnomAD) associated with Brugada syndrome are in strong LD and share haplotype block (according to SSLD). Heatmap generated with Haploview version 4.2. Color scheme based on 100× D’ values (values indicated unless D’ = 100), and log of the LOD ratios. Tracks: *cis*-regulatory regions (cantaloupe); common SNVs, according to 1KG (blue); and Brugada syndrome (BrS)-associated SNVs (red). Two SNVs (rs7430283 and rs73064548, shown as red lines in the blue track) reside in adjacent blocks from the rest, according to SSLD. Two SNVs (rs62242446 and rs62242447) adjacent to rs10428168 reside in the same block but are low frequency according to gnomAD (MAF < 5%), whereas common according to 1KG, thereby tested in Figure S2. A third SNV (rs62242448) adjacent to rs10428168, same block, only reaches near-significance in Figure 2D. (B) List of inferred haplotypes by cohort: Brugada syndrome-NFE (Hap1-8), 1KG-NFE (Hap1-5/9-11), Wellderly-NFE (Hap1-5/9/12), and GTEx-NFE (Hap1–5/9/10/13–15). Annotation: major, major alleles in all positions; Q-major, all but 1 major alleles; minor, minor alleles in all positions; Q-minor, all but 1 minor alleles; and mosaic, the rest. See also Figures S3A and S3B. (C) Scheme of the genotype phasing for the n = 86 Brugada syndrome cases. Analysis based on Beagle5.1 and SHAPEIT4 using Illumina short reads and validated by WhatsHap (version 0.18) using long-read sequencing of a 13-kb-long PCR fragment amplified from the DNA of each patient. Right, an example of data output is shown. (D and E) Frequency distribution of phased haplotypes in Brugada syndrome cases and control populations, as indicated. In (D) and (E), significance was tested by Fisher’s exact test. Results were considered significant (∗) if p values were below a Bonferroni-corrected α level of 3.33 × 10e−3 (0.05/15) based on the number of haplotypes tested. Iberian (IBS), British (GBR), Italian (TSI), and Utah residents of northern and western European descent (CEU). See also Figures S3C and S3D; Data S1, Table S5.

Next, we sought to determine whether a wide amplitude in association signals across the enhancer-containing pan-block could be explained by the participation of the underlying SNVs (rs1–7) in different allelic combinations, each having a distinct level of Brugada syndrome association. Assuming permutations of 7 SNVs with a major and a minor allele, there are n = 128 possible combinations.^2^ Of note, the rs1–7 alleles associated with Brugada syndrome are the major alleles in every case. However, as rs1–7 are in strong LD (D’ > 0.92; Figure 3A, LD heatmap), only a set of 5 or 6 common combinations is expected.^8^ To infer haplotype diversity in the Brugada cohort, we used 2 popular phasing algorithms, Beagle5.1^60^ and SHAPEIT4.^61^ Both algorithms inferred 8 haplotypes in the cohort, Hap1–8 (Figure 3B). Next, we sought to experimentally validate these haplotypes using Oxford Nanopore (long-read) sequencing technology.^62^^,^^63^ For this validation, we PCR amplified a 13-kb-long DNA fragment containing rs1–7 from each of the n = 86 Brugada syndrome cases (Figure 3C). After variant calling using the WhatsHap algorithm,^64^ we observed only n = 3 samples with discordant long-read sequencing and the 2 (short-read-based) predictions, which we removed from this point forward. Based on the remaining n = 83 Brugada syndrome cases, we validated Hap1–6 and Hap8. For Hap7 (inferred only in the removed samples), we chose short-read-based predictions, as long-read sequencing is more prone to errors than short-read sequencing.^65^

Likewise, we characterized the haplotype composition in the control groups. Using Beagle5.1 and SHAPEIT4, we found that the haplotype predictions were concordant between both algorithms in n = 177 Wellderly-NFE individuals (out of 196, or 90%). For the 1KG-NFE and GTEx-NFE datasets, phasing information is already available, and we could not use the gnomAD-NFE dataset from this point forward since individual-level genotypes are not available. Based on Wellderly, 1KG, and GTEx data, therefore, we could infer Hap1–5 and 7 additional allelic configurations, Hap9–15 (Hap9 imputed in the 3 controls, Hap10/11 imputed in 1KG-NFE and GTEx-NFE, Hap12 imputed in Wellderly-NFE, and Hap13–15 imputed in GTEx-NFE; Figure 3B). As expected,^8^ only 5 haplotypes were common (Hap1–5) in the enhancer pan-block, with an abundance >5% (Figure 3D). The rest, Hap6–15, represent rare combinations—with an abundance <1%, despite being constituted by SNVs with MAF ≥5% (Figure 3D). Hap1, Hap2, or Hap3, in particular, could be imputed in >98.5% of individuals in any dataset. Interestingly, furthermore, Hap1 and Hap3 are mutually exclusive allelic combinations, with Hap1 harboring the major allele in each rs1–7 position and Hap3 harboring the minor rs1–7 allele in each rs1–7 position (Figures 3B and S3A). The other 3 abundant haplotypes, Hap2/4/5, are “mosaic” combinations, with n = 4/3/2 major alleles of Hap1 and n = 3/4/5 minor alleles of Hap3, respectively (Figures 3B and S3A).^66^ Three rare haplotypes, Hap11/12/14, are also mosaics, while the rest, Hap6–9 and Hap10/13/15, are configurations differing in 1 allele from the all-major allelic combination, Hap1, or the all-minor allelic combination, Hap3. We refer to these rare combinations as quasi(Q)-major and Q-minor haplotypes, respectively (Figures 3B, S3A, and S3B).

Careful examination of the rs1–7 positions reveals that rs1/4/6 and rs2/3 are redundant if only the 5 abundant allelic combinations (Hap1–5) are considered. This fact explains in part the relative similarity of p values in the association signals for rs1/4/6, on the one side, and rs2/3, on the other side (Figure 2D). Still, at least 4 SNVs, rs1/2/5/7, are required to capture Hap1–5 diversity (Figure 3B), referred to as haplotype tag SNVs (htSNVs).^46^ If all haplotypes are considered, however, no particular SNV is fully redundant or a genuine htSNV (Figure 3B). This observation addresses the initial point of a wide dispersion of p values arising from the same block in case-control association studies and argues against a model of full redundancy for the 7 Brugada syndrome-associated SNVs in the enhancer pan-block. It also explains some differences in the level of association when comparing the Brugada cohort to different controls, as each control has a slightly different haplotype composition.

Next, we compared the haplotype frequencies between the Brugada and control groups. Hap1 is substantially more abundant in Brugada syndrome cases than controls—initially counterintuitive, as Hap1 harbors the major allele in each rs1–7 position (Fisher’s exact test: p = 6.86e−14, 1KG-NFE; p = 9.41e−14, Wellderly-NFE; and p = 5.63e−13, GTEx-NFE; Figure 3D; Data S1, Table S5). In contrast, Hap2 and Hap3 are significantly more common in controls than in cases (Fisher’s exact test: p = 9.25e−5 and p = 1.86e−6, 1KG-NFE; p = 1.07e−5 and p = 2.40e−5, Wellderly-NFE; and p = 1.59e−3 and p = 3.37e−7, GTEx-NFE, respectively; Figure 3D; Data S1, Table S5). To discard the possibility that these frequencies were the result of subjacent ancestry differences in NFE subpopulations, we compared haplotype frequencies between the Brugada cohort and four NFE subpopulations from the 1KG database: Iberian (IBS), British (GBR), Italian (TSI), and CEU. Hap1 abundance is still significantly higher and Hap2 and Hap3 abundance are still significantly lower in the Brugada group than in controls (Figures 3E and S3D; Data S1, Table S5). In combination, these analyses suggest that the 3 most common haplotypes in the NFE population (Hap1–3), surprisingly, show significantly different frequencies in Brugada syndrome cases.

### Recessive Hap1 inheritance associated with an increased risk for Brugada syndrome

**Figure 4:**
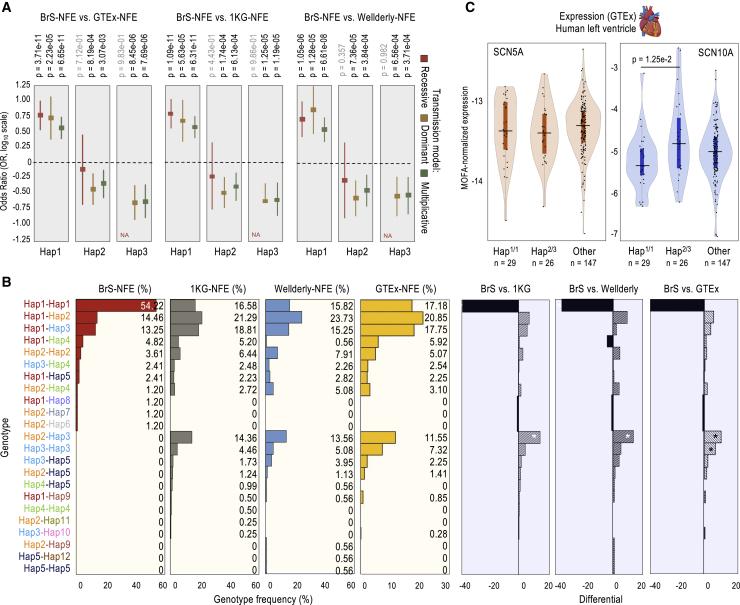
Transmission models, genotypes, and functional associations (A) Forest plot showing OR estimates and 95% confidence intervals (CIs) for the association between carrying the Hap1, Hap2, or Hap3 variants and risk of Brugada syndrome (BrS). ORs were obtained using logistic regression models assuming a recessive, dominant, or multiplicative inheritance after adjusting for gender. Horizontal lines indicate OR = 1. OR > 1 is associated with high risk of Brugada syndrome; OR < 1 is associated with lower risk of Brugada syndrome. Bonferroni-corrected p values indicated; significance threshold p < 3.33e−3, based on the number of total haplotypes tested (0.05/15). See also Data S1, Table S6. (B) Frequency of genotypes in the Brugada-NFE (n = 83), 1KG-NFE (n = 404), Wellderly-NFE (n = 177), and GTEx-NFE (n = 355) cohorts/datasets (percentages indicated), and differential frequency for the indicated comparisons. Significance tested by Fisher’s exact test. Results were considered significant (∗) if p values were below a Bonferroni-corrected α level of 1.79e−3 (0.05/23) based on the number of genotypes tested. See also Data S1, Table S6. (C) *cis-*eQTL analysis of the Hap^1/1^, Hap^2/3^, and the rest of genotypes (other), as in (B), using expression data of human left ventricle tissue generated by GTEx (no ancestry selection; MOFA-corrected expression). Violin plot shows median expression and box indicating interquartile range and sample point (number also indicated). Significance tested by 1-way ANOVA and Tukey honest significant differences (Tukey HSD) test for multiple pairwise comparisons. Significance threshold, p < 0.05. Significant case indicated. Rare genotypes only observed in the GTEx dataset (Hap^2/14^, Hap^3/14^, Hap^3/9^, Hap^1/13^, and Hap^1/15^) are not included. See also Figure S4.

We computed how the odds ratio (OR) estimates for Hap1–15 apply to 3 genetic transmission models—dominant, recessive, and multiplicative. The estimates reached significance only for Hap1–3; however, as the other 12 haplotypes are rare in the NFE population, their estimations should be taken cautiously (Figure 3D; Data S1, Table S6). For Hap1, the most significant models are recessive and multiplicative (Bonferroni corrected p values: p = 1.09e−11 and p = 6.31e−11 compared to 1KG-NFE; p = 1.05e−6 and p = 6.61e−8 compared to Wellderly-NFE; and p = 3.71e−11 and p = 6.65e−11 compared to GTEx-NFE; Figure 4A; Data S1, Table S6). In both cases, the OR values are >1 (OR = 6.28 and 3.81, respectively; Figure 4A, Hap1), consistent with a risk effect on the analysis of haplotype frequencies (Figures 3D and 3E, Hap1). Still, we favor the recessive over the multiplicative model as, while homozygous individuals (2 Hap1 copies) are overrepresented in the Brugada cohort (52.33%, compared to 14%–18% in controls; Figure 4B; Data S1, Table S7), the frequency of heterozygous individuals (1 Hap1 copy) is lower in the Brugada cohort than in the 3 control NFE populations analyzed (1KG-NFE, Wellderly-NFE and GTEx-NFE; Figure S3C). We suspect that the significance of the multiplicative model is artificially generated by an underrepresentation of non-Hap1 individuals in the Brugada cohort, which can be explained by the existence of the protective Hap^2/3^ genotype in the population (see below).

For Hap2 and Hap3, the most significant models are dominant and multiplicative (Figure 4A). For Hap2, in particular, we favor the dominant model, as we could infer Hap^2/2^ enrichment in the GTEx group and Hap^2/3^ is enriched in all controls (Figure 4A; Data S1, Table S7). We suspect that this effect is not significant in all Hap2 genotypes owing to the limited number of cases in this study (Figure 4B, differential panels). For Hap3, we could not favor a model over the other as we could not infer any Hap^3/3^ case in the Brugada cohort. We suspect that it would be necessary to analyze a larger cohort to reach a conclusion. For Hap2 and Hap3, in any case, the estimated OR values are markedly <1, supportive of a protective effect and consistent with the analysis of haplotype frequencies (Figure 4A; Data S1, Table S6). Notably, we could not infer any Hap^2/3^ case in the Brugada cohort, despite its high abundance in controls (11.55%–14.36%; Fisher’s exact test: p = 1.82e−5, p = 1.04e−4, and p = 2.08e−4 for 1KG-NFE, Wellderly-NFE, and GTEx-NFE; Figure 4B; Data S1, Table S7).

### Hap^1/1^/Hap^2/3^-based stratification reveals differential *SCN10A* but not *SCN5A* expression

Notably, Hap1 and Hap2 share 4 alleles (at rs2, rs3, rs5, and rs7 positions), which may pose doubts that these alleles confer causality (Figures 4B and S3A). However, they may confer this effect in combination with 1 or more of the other 3 alleles (at rs1, rs4, and rs6). Hap1 and Hap9 differ in only 1 allele (at rs1), and Hap^1/9^ is less abundant in Brugada syndrome cases than in controls (Figure 4B). This finding could be suggestive of rs1 contribution to the risk association (alone or in combination with the rest of the alleles). Hap9, however, is a rare haplotype, which prevents us from making a robust conclusion. Likewise, Hap1 and Hap8 differ in a single allele (at rs4). Hap^1/8^ is apparently as abundant in the Brugada and control groups, as opposed to Hap^1/1^ (Figure 4B). Once again, however, as we could infer only 1 Hap^1/8^ case (out of n = 83) in the Brugada cohort and none among the n = 936 controls, we avoided making a conclusion. We, therefore, sought to gain insights about the contribution of the individual alleles (at rs1–7) using unconditional logistic regression.

Comparing the 3 models of inheritance (dominant, recessive, and multiplicative), this analysis reveals no substantial differences between the predictive risk potential of rs1 and Hap1 (Data S1, Table S6). The same analysis indicates that genotyping rs4 or rs6 alone is almost as informative as genotyping rs1 or the haplotype, which is expected, as we concluded that rs1/4/6 are redundant in the context of the 5 common haplotypes, Hap1–5 (Data S1, Table S6). Genotyping rs2, rs3, rs5, or rs7 also serves as a relatively good predictor of Brugada syndrome risk, with significant p values ranging between p = 2.56e−5 and p = 8.46e−7 (Data S1, Table S6). In this case, however, it appears that, as a group, these SNVs are not major contributors to the association, as they can also be observed in homozygosis (as in Hap^1/1^) in Hap^1/2^ or Hap^2/2^ but without association to arrhythmia (Figure 4B). We propose at least 3 possible models of individual SNV contributions to Brugada syndrome risk. First, the 7 major alleles at rs1–7 contribute, as a group, to the risk. Second, only the major alleles at rs1/4/6 contribute to it. Third, only 1 of these 3 alleles (at rs1, rs4, or rs6) exerts the risk effect (as they are redundant in Hap1–5), whereas the major alleles at rs2/3/5/7 show risk association likely due to their frequent co-transmission with the major alleles at rs1/4/6. Regardless of the model, these analyses underscore the impact of haplotype composition in the control population to determine the strength of the association for individual SNVs, which confirms why the significance of some GWAS hits vary depending on the control population chosen.

Based on the opposite effects associated with Hap^1/1^ and Hap^2/3^, we next interrogated the correlation of these 2 genotypes and the individual alleles at rs1–7 with *SCN5A* and *SCN10A* expression. We postulated that the impact on gene expression (if any) may not be observed (or may be lower) in the case of the individual SNVs if more than 1 of the 7 alleles (at rs1–7) contribute to the phenotypic effects. To investigate this hypothesis, we compared *SCN5A* and *SCN10A* expression in adult human left ventricle samples segregated by genotype. For this test, we performed an expression quantitative trait loci analysis of local genomic effects (*cis*-eQTL) using genomic and gene expression data generated by the GTEx Consortium.^56^ To correct for potential technical confounders, we applied the multi-omics factor analysis (MOFA)^67^ (Figures S4A–S4C). As input to the model, we used RNA sequencing (RNA-seq) expression data of n = 202 left ventricle samples. After MOFA correction, we did not observe significant differences in *SCN5A* expression (Figure S4D). Likewise, we did not observe significant differences in *SCN10A* expression in the analysis of individual SNVs (Figure S4E). However, we did observe differences in *SCN10A* expression when comparing Hap^1/1^ and Hap^2/3^, while the rest of the genotypes correlated with intermediate gene expression (Figure 4C). We suspect that, as some major alleles are shared between Hap1 and Hap2, some effects (risk, protective, or non-risk/non-protective) may be mixed when considering individual SNVs, diminishing their predictive value on gene expression. These analyses suggest that haplotypes, as genotype combinations, are better predictors of *SCN10A* expression than individual SNVs, when using the same corrections. In addition, we did not observe significant differences in the severity of the symptoms among Brugada syndrome cases segregated by the Hap^1/1^ and the rest of genotypes. However, we detected a general trend of higher numbers of Brugada syndrome cases with severe disease among Hap^1/1^ carriers. The percentage of Brugada syndrome cases with familial history of sudden death was also significantly higher in Hap^1/1^ than in the other genotypes (Data S1, Table S8).

### Hap^1/1^ and Hap^2/3^ are common in some but not all human super-populations

**Figure 5:**
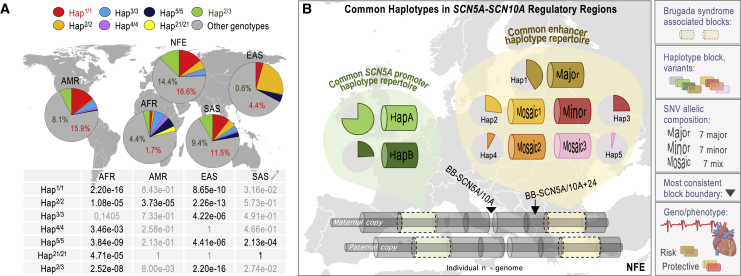
Worldwide genotype frequencies at the enhancer-containing pan-block (A) Frequency of the risk (Hap^1/1^), protective (Hap^2/3^), and multiple homozygote genotypes inferred worldwide using the 1KG Phase 3 database, (n = 404 NFE; n = 347 AMR; n = 661 AFR; n = 489 SAS; and n = 504 EAS individuals). Table depicts p values for the comparisons of the inferred frequencies between NFE and each of the rest of the human super-populations. Results were considered significant (∗) if p values were below a Bonferroni-corrected α level of 7.1e−3 (0.05/7) based on the number of genotypes tested. (B) Haplotype pan-block structure and common haplotype composition in haplotype pan-blocks containing *cis*-regulatory regions across the *SCN5A-SCN10A* locus. Haplotypes at the enhancer-containing pan-block based on this study and haplotypes at the promoter-containing pan-block based on a prior study.^31^ See text for details.

Epidemiologic studies have shown differences in the prevalence of Brugada syndrome worldwide.^68^ We wondered, therefore, whether the distribution of the risk and protective genotypes varies across human super-populations. For simplification, we focused on Hap^1/1^, Hap^2/3^, and the homozygote genotypes inferred in at least 1 super-population (i.e., Hap^2/2^, Hap^3/3^, Hap^4/4^, Hap^5/5^, and Hap^21/21^ [the latter is common in the AFR super-population]; Figures 5A and S3A). We observed that Hap^1/1^ and Hap^2/3^ are almost as abundant in AMR and SAS as in NFE (11.5%–16.5% and 8.1%–14.4%, respectively; Figure 5A), while these genotypes are significantly less abundant in EAS and AFR (Figure 5A). Moreover, while the 7 SNVs (rs1–7) in the haplotypes are in strong LD in NFE, AMR, and SAS (D’ > 0.92), they are not in the other 2 super-populations, especially in EAS (Figure S5). This analysis agrees with the block-partitioning predictions shown in Figure 1 (colored tracks), inferring disparity in haplotype-block frameworks across super-populations. We also note a low frequency of the Hap^1/1^ genotype and a high frequency of mosaic haplotypes in the AFR super-population, in agreement with a study describing that haplotypes as Hap1 exhibit statistically significant avoidance in the African continent.^66^ However, in regard to comparing super-populations, we acknowledge that moving from a haplotype definition in NFE to the rest of the super-populations demands some precautions, as the depth of the analysis of haplotype diversities and haplotype structures is different (Figures 1 and S1).

## Discussion

This study provides supporting evidence on the importance of fine-mapping clusters of GWAS hits to correctly annotate disease-associated variants. We reached this conclusion through the analysis of the *SCN5A-SCN10A* locus, repeatedly associated with multiple ECG traits and cardiac conduction disorders^1^. In this locus, we have annotated 2 haplotype blocks that accumulate most of the common SNVs associated with Brugada syndrome, either by genetic profiling or GWASs, and we refer to them as the promoter and the enhancer blocks, respectively (Figure 5B). Based on a previous GWAS,^34^ only the enhancer block accumulates a cluster of hits associated with this condition in individuals with European ancestry, while the common SNVs associated with ECG traits in the *SCN5A* promoter were described in the Asian population.^31^ In support that the enhancer block acts as a heritable unit, we found that 7 of the 10 common SNVs examined in the block are associated with Brugada syndrome and an eighth SNV is associated at subthreshold levels. However, their individual association signals are rather dispersed, which led us to deconvolute the underlying haplotype composition. Interestingly, we found that >98% of individuals with NFE ancestry carry at least 1 of 3 highly common haplotypes, Hap1–3, and ∼80% carry only Hap1–3 (i.e., Hap^1/1^, Hap^1/2^, Hap^1/3^, Hap^2/2^, Hap^2/3^, or Hap^3/3^). Hap1 and Hap3 represent non-overlapping combinations, and Hap2 is a Hap1/Hap3 mosaic. Mutually exclusive allelic combinations are known as yin-yang pairs.^69^^,^^70^ Yin-yang pairs are not unusual in the human population.^66^ However, the model of selection that led to such high enrichment of Hap1 and Hap3 during the history of the human population is intriguing. Notably, Hap1–3 are associated with Brugada syndrome, with more than half of the cases carrying the Hap^1/1^ genotype, whereas none of the cases carries the Hap^2/3^ genotype. OR estimates are suggestive of risk and protective effects, respectively. Arguably more intriguing, Hap1 and Hap2 share 4 alleles, the major allele at rs2, rs3, rs5, and rs7. Their risk/protection effects, therefore, depend on haplotype and genotype context. These alleles are associated with Brugada risk in Hap^1/1^, with non-risk in the heterozygous Hap^1/−^ genotype, and with protection in Hap^2/3^. Meanwhile, the risk associated with the other 3 major alleles in Hap1 (at rs1, rs4, and rs6) will only be relevant in the context of the Hap^1/1^ genotype (although it is unclear whether they would also be relevant in the case of the Hap^1/7^ or Hap^7/7^ genotypes [not observed in our analyses]). In combination, our analyses corroborate previous work suggesting that haplotypes outperform the value of single SNVs to understand the genetics underlying disease,^71^ also indicating the critical importance of deconvoluting clustered GWAS information and revealing the risks of indiscriminately implying SNV redundancy on clustered GWAS data.

About Brugada syndrome genetics, we made three important observations. First, the risk haplotype, Hap1, would fit with a model of recessive inheritance. At present, the most widely accepted transmission model for Brugada syndrome is autosomal dominance (likely, as this model is more easily detected in GWASs than the recessive option). Having said that, two recent reports also suggest a recessive model of inheritance for Brugada syndrome: a *TRPM4* null variant in homozygosis^72^ and variants in the X-linked *KCNE5* gene.^73^ The recessive model is not unusual for non-coding pathogenic variants.^74^ Notably, the Hap^1/1^ genotype is uncommon in the EAS super-population, which is consistent with epidemiologic studies showing that Brugada syndrome cases across the Chinese, Japanese, Taiwanese, and South Korean populations carry fewer deleterious variants than Caucasians in the *SCN5A* locus.^68^ A second relevant observation is the identification of a Brugada-protective non-coding genotype based on haplotypes, Hap^2/3^. Prior studies report protective variants against arrhythmia in the coding regions of the *KCNQ1* and *SCN5A* genes^26^^,^^75^ and protective variants against Brugada syndrome in a coding region of the *SCN10A* gene and in a non-coding site downstream the *HEY2* gene in the Japanese population.^19^^,^^76^ The third relevant observation is that the Hap^1/1^ and Hap^2/3^ genotypes are anti-correlated with *SCN10A* but not *SCN5A* expression in the adult left ventricle. A previous report suggested that rs6801957 in homozygosis (rs1 in our study) is associated with decreased *SCN5A* expression.^41^ Another study suggested that rs6801957 modulates enhancer activity in a cell reporter assay.^35^ It is unclear, however, whether these prior observations were obtained in the context of the Hap^1/1^ genotype.

Our findings will contribute to the debate over the role of Na_V_1.8 (encoded by *SCN10A*) in cardiac conduction and its expression in cardiac myocytes. Na_V_1.8 was originally reported in nociceptive sensory neurons.^77^ Later, the identification of an intronic region as a major risk region for prolonged QRS duration led to follow-up studies indicating that Na_V_1.8 contributes to cardiac electrophysiology.^78^^,^^79^ It is worth noting that an exonic SNV in the *SCN10A* gene, rs6795970, carries a protective (G) or a risk (A) allele at position 1,073 of the protein, in LD with rs6, rs10428132, in this study.^19^^,^^34^

In conclusion, our findings provide evidence of the importance of deconstructing clustered GWAS hits in the context of haplotype blocks and haplotype composition and encourage the re-analysis of published GWAS data and reconsideration of future analytical decisions in the GWAS field. For Brugada syndrome cases, in particular, we describe that carrying 2 Hap1 copies in the enhancer-containing haplotype block is, potentially, the most common risk genotype (Hap^1/1^) in individuals of non-Finnish European ancestry, while carrying 1 Hap2 copy and 1 Hap3 copy (Hap^2/3^) would be the most common protective genetic feature.

### Limitations of study

Our study has limitations. First, it is based on a relatively small number of cases that we pre-selected for not carrying rare deleterious variants in the coding regions of the *SCN5A* gene (the latter decision may have helped us to identify the link of Hap^1/1^ and Hap^2/3^ to Brugada syndrome). Second, it is based on SNVs overlapping some chromatin features in cultured human cardiac myocytes, while other functionally relevant regions are also likely to exist in these or other heart cells. Third, we cannot exclude the possibility that rare variants co-evolving with Hap1 are the ultimate contributors to Brugada syndrome. For example, recent publications suggest the possibility of an interplay between rare and common variants.^80^, ^81^, ^82^ A model has also been proposed in which rare SNVs generate a “synthetic association” by occurring more frequently near one allele than another.^83^ In this case, however, we would find it intriguing that the Hap^1/−^ genotypes are not significantly associated with Brugada syndrome. Non-coding variation may also contribute to disease by modifying the penetrance of exonic variants, known as variable penetrance.^84^ In this sense, the exonic SNV in the *SCN10A* gene, rs6795970, can lead to a missense allele (alanine to valine) and is in LD with the common SNV rs10428132 (rs6, in this study)^19^^,^^34^ in the enhancer-containing block (Figure 1B). With regard to exonic variants, it is also important to highlight that synonymous mutations—currently not considered as deleterious—may have an impact on mRNA stability, expanding the repertoire of possible combined effects between coding and non-coding SNVs.^85^ In general, the functional validation of our findings will be challenging. We would like to stress, for example, that validating a functional role for Hap1–3 on *SCN10A* expression will require the identification of the *SCN10A*-expressing heart cell, while it is still a matter of debate as to the identity of this cell. Also, even if *SCN10A* expression could be properly modeled, if the goal is to test haplotype effects, then genome editing must be applied to every single SNV in the haplotypes. Otherwise, any effect attributed to an edited SNV could also be attributed to the haplotype. Finally, recent data reveal the widespread presence of regulatory RNA elements in intronic sequences that function in the context of the transcriptome.^86^ Thus, it must be considered that there may be functional SNVs in LD with the 7 SNVs in Hap1–3 that do not belong to *cis*-regulatory elements and have a functional impact at the level of the transcriptome.

## STAR★Methods

### Key resources table

**Table:** 

REAGENT or RESOURCE	SOURCE	IDENTIFIER
**Biological samples**		

DNA from 86 Brugada syndrome patients	This paper	N/A

**Chemicals and reagents**		

Supreme NZYLong DNA polymerase	NZYTech	MB331
dNTPs NZYMix	NZYTech	MB086
Pure IT ExoZap	Ampliqon	A620601
Agencourt AMPure XP Beads	Beckman Coulter	A63881
LongAmp Taq 2X Master Mix	NEB	M0287
Short read eliminator XS	Circulomics	SKU SS-100-121-01
NEBNext Companion Module for Oxford Nanopore Technologies Ligation Sequencing	NEB	E7180
NEBNext Quick Ligation Module	NEB	E6056

**Critical commercial assays**		

Nextera Rapid Capture Custom Enrichment Kit	Illumina	FC-140-1008
PCR Barcoding Expansion Pack 1-96	Oxford Nanopore Technologies	EXP-PBC096
Oxford Nanopore 1D DNA ligation Sequencing Kit	Oxford Nanopore Technologies	SQK-LSK109
Flow Cell Priming Kit	Oxford Nanopore Technologies	EXP-FLP002

**Deposited data**		

Raw and analyzed data	This paper	EGAS00001004927
Scripts	This paper	https://github.com/Mel-lina/Brugada
Script for CTCF-motif orientation analysis	This paper	https://github.com/bdolmo/DeepBindTK
ChIP-seq data for DHS from HCMs	Encode Project Consortium^43^	GSM736516 and GSM736504
ChIP-seq data for H3K4me3 from HCMs	Thurman et al.^44^	GSM945308
ChIP-seq data for CTCF from HCMs	Wang et al.^45^	GSM1022657 and GSM1022677
Hi-C-seq data from hESCs	Dixon et al.^47^	GSM862723 and GSM892306
WGS genotypes from Human subjects	Erikson et al.^55^	RRID:SCR_011519
WGS genotypes from Human subjects	1000 Genomes Project Consortium et al.^39^	RRID:SCR_006828
WGS genotypes from Human Subjects	GTEx Consortium et al.^56^	RRID:SCR_013042
Illumina OMNI 2.5 genotypes from Human subjects	10000 Genome Project Consortium et al.^39^	RRID:SCR_006828
WGS genotypes from Human subjects	Karczewski et al.^54^	RRID:SCR_014964
RNA-Seq data from Human Heart Left Ventricle samples	GTEx Consortium et al.^56^	RRID:SCR_013042
Phased genotypes from Human subjects	GTEx Consortium et al.^56^	RRID:SCR_013042
Low complexity regions in the human genome GRCh37/hg19	Li^87^	Li, 2014^87^
GATK Bundle for human reference genome hg19	Broad Institute	gsapubftp-anonymous@ftp.broadinstitute.org

**Oligonucleotides**		

Tailed forward primer for long-range PCR: TTCTGTTGGTGCTGATATTGCGCCATGACCA TTGTTATTTGTCCAGA	This paper	N/A
Tailed reverse primer for long-range PCR: ACTTGCCTGTCGCTCTATCTTCCCTGAAG AAATGTCACGGCTTGTTAG	This paper	N/A
Tailed forward primer for nested long-range PCR: TTCTGTTGGTGCTGATATTGCAGTAACTGAA AATGCTTCTGAGTGGC	This paper	N/A
Tailed reverse primer for nested long-range PCR: ACTTGCCTGTCGCTCTATCTTCTTGAAGAG AGGTGACAAAACAAAGGG	This paper	N/A

**Software and algorithms**		

HOMER	Benner at al.^11^	RRID:SCR_010881
DesignStudio™	Illumina	https://www.illumina.com/products/by-type/informatics-products/designstudio.html
Skewer v0.1.123	Jiang et al.^88^	RRID:SCR_001151
BWA-MEM v0.7.17	Li and Durbin^89^	RRID:SCR_010910
SAMtools v1.3.1	Li et al.^90^	RRID:SCR_002105
Picard v2.18.9	Broad Institute	RRID:SCR_006525
GATK v3.8-0	Mckenna et al.^53^	RRID:SCR_001876
PLINK v1.90b6.9	Purcell et al.^91^	RRID:SCR_001757
Rtsne	Van der Maaten and Hinton^92^	RRID:SCR_016342
HaploView v4.2	Barrett et al.^40^	RRID:SCR_003076
Beagle5.1	Browning et al.^60^	RRID:SCR_001789
SHAPEIT4	Delaneau et al.^61^	https://odelaneau.github.io/shapeit4/
Guppy v3.2.10	Oxford Nanopore Technologies	https://nanoporetech.com/
minimap2 v2.17	Li^93^	RRID:SCR_018550
WhatsHap v0.18	Patterson et al.^64^	https://whatshap.readthedocs.io/en/latest/
R v3.5.2	R Project	https://www.r-project.org/
ggplot2	R Project	https://cran.r-project.org/web/packages/ggplot2/index.html
IGV	Broad Institute	RRID:SCR_011793
DESeq2	Love et al.^67^	RRID:SCR_015687
MOFA+	Argelaguet et al.^67^	https://biofam.github.io/MOFA2/

**Other**		

MinION Flow Cell R9	Oxford Nanopore	FLO-MIN106D

### Resource availability

#### Lead contact

Further information and requests for resources and reagents should be directed to and will be fulfilled by the Lead Contact, Sara Pagans (sara.pagans@udg.edu).

#### Materials availability

This study did not generate new unique reagents.

#### Data and code availability

Scripts generated in this study are available from https://github.com/Mel-lina/Brugada and https://github.com/bdolmo/DeepBindTK. The sequencing data generated in this study is found at European Genome-Phenome Archive with accession number EGAS00001004927. Publicly accessed datasets: DNaseI-seq for DHS (GSM736516 and GSM736504), H3K4me3 ChIP-seq (GSM945308), and CTCF ChIP-seq (GSM1022657 and GSM1022677) datasets were generated in human cardiac myocytes and downloaded from NCBI-GEO (https://www.ncbi.nlm.nih.gov/geo);^43^, ^44^, ^45^ and, Hi-C-seq data (GSM862723 and GSM892306) for TAD analysis.^47^

### Experimental model and subject details

#### Subject details

##### Brugada syndrome cohort

We recruited a group of unrelated individuals with Brugada syndrome who were diagnosed based on a pattern of “coved” ST-segment elevation on the right precordial ECG leads (type I ECG pattern), either at baseline or after pharmacological induction using ajmaline or flecainide treatment. Gender, age at diagnosis and clinical characteristics are described in Table 1. Peripheral blood from each patient was collected in 4 mL EDTA Anti-Coagulant BD Vacutainer tubes and genomic DNA was extracted using the Chemagic MSM I Instrument (PerkinElmer) following manufacturer’s recommendations. DNA was eluted in 300 μL of Elution Buffer (EB, QIAGEN) and stored at −20°C until used. After, (1) analysis of *SCN5A* exonic regions based on Sanger sequencing using the 3130XL Genetic Analyzer Instrument (Applied BiosystemsTM) or based on *SCN5A*-targeted NGS using the MiSeq Instrument (Illumina), and (2) ancestry analysis based on targeted NGS sequencing of *cis*-regulatory regions (see “Capture and sequencing of cis-regulatory regions from Brugada syndrome cases” in the Method details section), we selected n = 86 cases who do not harbor deleterious variants in the major Brugada syndrome-related gene *SCN5A* and that have a NFE ancestry (see “Ancestry admixture analysis” in Method details). Deleteriousness of *SCN5A* variants was interpreted following the standards and guidelines from the American College of Medical Genetics and Genomics (ACMG).^94^ Population-based frequency of annotated *SCN5A* variants was obtained from the Exome Variant Server^95^; while the *in silico* deleteriousness prediction was computed using the Protein Variation Effect Analyzer (PROVEAN),^96^ MutationTaster2,^97^ and the Polymorphism Phenotyping v2 (PolyPhen-2).^98^

This study was conducted according to the Declaration of Helsinki Principles and complies with the European and National Code of Practice. All Brugada syndrome cases, recruited at the Clinic Hospital of Barcelona and the Dr. Josep Trueta Hospital of Girona, signed an informed written consent to participate in the study. This study was approved by the Clinical Research Ethics Committee of the Dr. Josep Trueta Hospital (#2012.097).

##### Wellderly cohort

The Wellderly dataset contains whole-genome sequencing (WGS) data of n = 1,354 healthy-aging individuals who, at the time of recruitment, were >80 years old with no history of chronic disease nor history of taking chronic medications.^55^ In this study, we selected n = 196 individuals from the Wellderly cohort who satisfied two criteria: (1) individuals genotyped using Illumina chemistry (n = 200 out of 1,354), as we used for Brugada syndrome samples, thus avoiding any technical biases; and, (2) individuals with a NFE ancestry (n = 196 out of the 200; see “Ancestry admixture analysis” in Method details). Individual-level genotype information in VCF format relative to the GRCh37/hg19 reference genome was generously provided by Dr. Eric Topol and Dr. Manuel Rueda.^55^

##### GTEx database

The Genotype-Tissue Expression (GTEx) dataset was previously described.^56^ We accessed this dataset through NCBI dbGaP (the Database of Genotypes and Phenotypes), deposited under the accession code phs000424.v7.p2 (approved access #82151, May/2020). For SNV analyses, we used high-coverage (30X) Illumina WGS data of n = 652 unrelated individuals in the GTEx dataset. This cohort consisted of n = 561 individuals with self-reported European ancestry, n = 75 of African ancestry, and n = 8, n = 3 and n = 5 of Asian, Amerindian, and unknown ancestry, respectively. For each sample, we downloaded a VCF file containing genotype calls relative to the GRCh37/hg19 reference genome. For haplotype analyses, we used the data of n = 355 individuals, which corresponds to those with NFE ancestry (see “Ancestry admixture analysis” in Method details) and phasing information available. Phased genotypes from GTEx samples (obtained using Illumina Omni genotyping arrays) were downloaded from the accession code phs000424.v7.p2.^56^

##### 1000 Genomes (1KG) Phase 3 datasets

The 1KG Project was previously described.^38^^,^^39^ Individual-level genotype data of n = 2,405 individuals from five human super-populations, obtained by Illumina sequencing, was downloaded in VCF format from the 1KG Project Phase 3 (v5a, ftp://ftp.1000genomes.ebi.ac.uk/vol1/ftp/release/20130502/). The 1KG study consisted of n = 661 individuals with African ancestry (AFR), n = 347 of American ancestry (AMR), n = 504 of East-Asian ancestry (EAS), n = 404 of non-Finnish European ancestry (NFE), and n = 489 of with South-Asian ancestry (SAS). For each sample, we downloaded VCF files containing genotype calls relative to the GRCh37/hg19 reference genome, which we used for SNV analyses. We also downloaded individual-level genotypes from n = 2,218 individuals from five human super-populations, obtained with the Illumina Omni 2.5 genotyping array, which we used for the ancestry admixture analysis of GTEx individuals. The 1KG Illumina Omni 2.5 cohort consisted of n = 542 individuals with AFR ancestry, n = 426 of AMR ancestry, n = 588 of EAS ancestry, n = 549 of NFE ancestry, and n = 113 of SAS ancestry (ftp://ftp.1000genomes.ebi.ac.uk/vol1/ftp/release/20130502/supporting/hd_genotype_chip/).

##### gnomAD dataset

The Genome Aggregation Database (gnomAD) was previously reported.^54^ It contains WGS data from n = 15,708 unrelated individuals aligned to the GRCh37/hg19 human reference genome (dataset v2.1.1).^54^ Population-level allelic frequencies are available for each variant, but not individual-level genotypes. For our study, we downloaded VCFs from the gnomAD repository (https://gnomad.broadinstitute.org/downloads) and extracted allele frequencies only from the subset of n = 7,718 NFE individuals present in the dataset.

### Method details

#### Patterns of LD and haplotype-block structure

Haplotype blocks for *SCN5A-SCN10A* locus (chr3:38,516,506-38,841,720) in n = 404 NFE; n = 661 AFR; n = 347 AMR; n = 489 SAS and, n = 504 EAS individuals from 1000 Genomes Phase 3 were defined using HaploView (v4.2).^40^ HaploView incorporates several algorithms for haplotype partitioning: the Confidence Interval (CIT),^6^ the Four Gamete Rule (FGR),^99^ and the Solid Spine of Linkage Disequilibrium (SSLD).^40^ Given that each method differs greatly from the others in its scope of the definition of the haplotype blocks, we obtained haplotype block estimates using all three partitioning methods on default settings. Pan-blocks were annotated by aggregating block-boundary coordinates according to each haplotype partitioning method. The coordinates found in at least ten cases (out of a total of fifteen, considering five super-populations and three partitioning methods) were defined as pan-boundaries as they represent the subset of regions that most frequently were predicted as haplotype block boundaries considering the five super-populations and three partitioning methods. Pan-blocks represent the regions between pan-boundaries.

#### CTCF-motif orientation analysis

We predicted the orientation of the CTCF motif at CTCF-occupied sites in the *SCN5A-SCN10A* locus (chr3:38,516,506-38,841,720) using an in-house-developed tool consisting on a Perl wrapper around DeepBind (script available at https://github.com/bdolmo/DeepBindTK). DeepBind is a deep-learning algorithm that we implemented using the CTCF model (downloaded from http://tools.genes.toronto.edu/deepbind/).^100^

#### Annotation of *cis*-regulatory regions in Brugada syndrome-associated loci

For genotyping, we annotated a set of candidate *cis-*regulatory regions across the *SCN5A*-*SCN10A* locus and five additional Brugada syndrome-associated loci, *SCN2B, SCN3B, CACNA1C, CACNB2* and *CACNA2D1*.^49^, ^50^, ^51^, ^52^ As aggregate annotations based on international consortia were not yet available at the time of this design, at least to our knowledge, we annotated these genomic regions using maps of open-chromatin regions (DHS), CTCF binding, and H3K4me3 accumulation in human cardiac myocytes.^43^, ^44^, ^45^ We defined the limits of the annotations to the upstream (5′) and downstream (3′) boundaries of the TAD adjacent to the TAD containing the *SCN5A-SCN10A* locus (i.e., the annotation included three TADs for each locus, ∼4-7 Mb in total). The exact positions of the boundaries were previously defined based on Hi-C-seq data in human embryonic stem cells,^47^ relative to the human reference genome GRCh37/hg19. The regions were annotated using a well-established pipeline of ChIP-seq data analysis, HOMER, as previously described.^88^, ^101^, ^102^, ^103^, ^104^ After consolidation of the three sets of coordinates underlying DHS, CTCF-occupied sites, and H3K4me3 peaks, we obtained n = 1,291 regions. We added two 300 bp-long regions to this set whose coordinates were centered to the two lead SNVs previously reported in the *SCN5A-SCN10A* locus, rs10428132 and rs11708996, associated with Brugada syndrome.^34^ We had to manually add these two regions as they did not overlap with the annotated DHS, CTCF-occupied sites, and H3K4me3 peaks. In total, therefore, we annotated n = 1,293 regions for deep genotyping and the coordinates can be found in Data S1, Table S2.

#### Capture and sequencing of *cis*-regulatory regions from Brugada syndrome cases

The capturing of the n = 1,293 targeted regions in our cohort of Brugada syndrome cases was performed using n = 5,546 probes (80-mer) designed using the web-based tool DesignStudio™ from Illumina. We set the probes to be non-overlapping with a standard center-to-center spacing between adjacent probes of an average of n = 230 bp (library size validated using a 2100 BioAnalyzer Instrument, Agilent). For each Brugada syndrome individual, we prepared a DNA library for deep NGS of the n = 1,293 regions using the Nextera Rapid Capture custom enrichment kit (NRC; Illumina). We followed the NRC Enrichment Reference Guide with a minor adaptation during DNA tagmentation. Specifically, we fragmented DNA with 25 μL of Tagment DNA Buffer, 10 μL of Tagment DNA Enzyme 1, and 5 μL of nuclease-free water for 10 min at 58°C. Brugada DNA samples were fragmented and uniquely barcoded for paired-end sequencing using NRC index 1 and index 2 primers. Indexed samples were combined for multiplex sequencing in pools of up to n = 12 samples, as indicated in the NRC guide. Final DNA libraries were normalized to 2-3 nM and sequenced on the Illumina HiSeq2500 sequencer using a 100-base paired-end double index read format at the Center for Genomic Regulation (CRG, Barcelona, Spain). We obtained an average of 5.9 million reads per sample, from which 3.8 million ± 948,849 corresponded to the regions of interest (target enrichment of 64.79%).

#### Read alignment and variant calling of short reads

FASTQ files were pre-processed with Skewer (v0.1.123)^89^ to remove NRC adaptor sequences added during library preparation. Read ends were quality-pruned using an in-house Perl script and aligned to the human reference genome GRCh37/hg19 using the Burrows-Wheeler Aligner (BWA-MEM; v0.7.17)^105^ under default settings. Reads with ambiguous multiple secondary alignments were removed using Sequence Alignment/Map tools (SAMtools; v1.3.1)^90^ and sequencing duplicates were removed using Picard (v2.18.9). Variant calling was performed using the Genome Analysis Toolkit (GATK; v3.8-0)^53^ following its best practices recommendations.^106^ Briefly, we left-aligned indels using the LeftAlignIndels, we generated genotype VCFs for each Brugada syndrome sample with the HaplotypeCaller and, we merged all genotype VCFs into a joint VCF using the GenotypeGVCFS. Finally, we refined the joint VCF to reduce the number of false positives by applying the GATK variant quality score recalibration (VQSR). For VQSR, we built an adaptive error model based on multiple parameters from public resources of known variation, downloaded from GATK Bundle for the human reference genome hg19 (https://gatk.broadinstitute.org/hc/en-us/articles/360035890811-Resource-bundle) (Data S1, Table S3). Variants labeled as low quality by the VQSR, variants with missing genotypes in any Brugada syndrome case, variants falling within low-complexity regions extracted from^107^ and ambiguous indels (i.e., multiallelic indels) were removed from further analysis (Data S1, Table S3). Final variants were annotated with the gnomAD database to extract variant identity (rs) and allele frequencies for NFE individuals.

#### Ancestry admixture analysis

Ancestry admixture analysis of Brugada syndrome, Wellderly and GTEx individuals was performed using PLINK (v1.90b6.9).^87^ For Brugada syndrome and Wellderly individuals, we used Illumina sequencing-based 1KG genotypes as a reference panel. In this case, as we only had genotype data from the n = 1,293 genotyped regions, the ancestry admixture analysis was restricted to the common biallelic SNVs (MAF ≥ 5%) found within these regions. To minimize the effect of LD in the analysis, we pruned markers in high LD (with the --indep-pairwise 50 10 0.2 option in PLINK). This resulted in a collection of n = 1,151 ancestry informative SNVs that was input to PLINK for a joint PCA of 1KG Illumina-based sequencing, Brugada syndrome and Wellderly samples. The first 6 principal components were extracted and input to t-Distributed Stochastic Neighbor Embedding (t-SNE).^91^ We ran t-SNE under its R implementation (Rtsne)^91^ with the following parameters: perplexity of 30, default learning rate of 200, default maximum number of iterations of 1,000 and default exaggeration factor of 12. Ancestry admixture results for Brugada syndrome and Wellderly individuals obtained with Rtsne were visualized through the R package ggplot2. We excluded from further analysis n = 4 Wellderly individuals for not having a NFE ancestry. For GTEx individuals, we used Illumina Omni 2.5 array-based 1KG genotypes to infer their ancestry admixture. In this case, as we had genotypes from WGS data, we extended the ancestry admixture analysis to n = 6,248,213 biallelic SNVs with MAF ≥ 5%, which were LD-pruned using the --indep 50 10 2 option in PLINK. This resulted in a collection of n = 727,181 ancestry informative SNVs that was input to PLINK for a joint PCA of 1KG Omni 2.5 and GTEx samples. Ancestry admixture results for GTEx individuals obtained with PLINK were visualized through the R package ggplot2. We excluded from further analysis n = 91 GTEx individuals for hot having a NFE ancestry.

#### Association analysis of common and low-frequency variants

To test the association of common (MAF ≥ 5%) and low-frequency SNVs (MAF = 0.5%–5%) with Brugada syndrome, we performed a case-control association analysis comparing the n = 86 Brugada syndrome cases with a control group of n = 7,718 unrelated NFE-ancestry individuals from gnomAD. For confirmatory purposes of common SNVs, we performed three additional case-control association analysis comparing the Brugada syndrome cases with a control group of: (1) n = 404 unrelated NFE-ancestry individuals from 1KG (1KG-NFE), (2) n = 196 unrelated NFE-ancestry individuals from the Wellderly cohort (Wellderly-NFE), and (3) n = 355 unrelated NFE-ancestry individuals from GTEx. All association analyses were performed using a Fisher’s exact test. Common SNVs were considered associated with Brugada syndrome if the p value was less than the Bonferroni corrected α level of 2.36e-05 (0.05/2,121), based on the number of variants tested. Similarly, low-frequency SNVs were associated with Brugada syndrome if the p value was less than the Bonferroni corrected α level of 4.06e-05 (0.05/1,232).

#### Haplotype phasing

Haplotype phase for the enhancer block rs1-7 for the n = 86 Brugada syndrome cases was estimated from both short-read and long-read sequencing data. For short-read sequencing data, we used Beagle5.1^60^ and SHAPEIT4^61^ on a reference-based mode using human genetic maps of recombination. The reference panel from NFE individuals was downloaded from 1KG Phase 3. HapMap genetic maps were downloaded from http://bochet.gcc.biostat.washington.edu/beagle/ for Beagle and from https://github.com/odelaneau/shapeit4/tree/master/maps for SHAPEIT. For long-read sequencing data, we used WhatsHap (v0.18)^64^ following the recommended workflow. Haplotypes for each Brugada syndrome case were individually estimated by running WhatsHap under the *phase* subcommand and setting the –tag option to PS (phase). As input, we used the short-read sequencing VCF file of a given Brugada syndrome case and the pre-processed long-read sequencing BAM from that same Brugada syndrome case. To visualize phased haplotypes, we ran WhatsHap under the *haplotag* subcommand using, as input, the WhatsHap phased VCF of a Brugada syndrome case and the pre-processed long-read sequencing BAM from that same Brugada syndrome case. *Haplotag* created a new BAM file with tagged reads according to their estimated haplotype. For visualization purposes, we opened the haplotagged BAMs with IGV and we grouped long-read alignments by HP tag (haplotype). We only accepted for further analysis Brugada syndrome cases with the same haplotype phase estimation in at least two of the three phasing tools used (Beagle5.1, SHAPEIT4 and WhatsHap). For the Wellderly samples, haplotype phase estimates were generated using Beagle5.1 and SHAPEIT4, as described above. We only accepted for further analysis Wellderly individuals with the same haplotype phase estimation by Beagle5.1 and SHAPEIT4.

#### Oxford Nanopore long-read sequencing using the MinION device

From each Brugada syndrome genomic DNA sample, we amplified by PCR the *SCN10A* haplotype region containing rs1-7. We used the standard protocol of the Supreme NZYLong DNA polymerase (5 U/μL, NZYTech): 5 μL of 10X Reaction Buffer, 5 μL of dNTP mix (2.5 mM each), 1.75 μL of forward primer 5′-TTCTGTTGGTGCTGATATTGCGCCATGACCATTGTTATTTGTCCAGA-3′ (10 nM), 1.75 μL of reverse primer 5′-ACTTGCCTGTCGCTCTATCTTCCCTGAAGAAATGTCACGGCTTGTTAG-3′ (10 nM), 300 ng of genomic DNA, 1 μL of Supreme NZYLong DNA polymerase and PCR grade water up to 50 μL. PCR cycling conditions were: 94°C for 5 min, followed by 30 cycles of 94°C for 20 s, 60.5°C for 30 s, and 68°C for 14 min followed by a final step of 68°C for 21 min and hold at 4°C. PCR products showing low DNA concentration were cleaned with ExoSap-IT (ThermoFisher) and subjected to a second amplification round (nested PCR). Nested PCR was performed with 1 μL of cleaned PCR product (at 1/500 dilution) following the same PCR protocol as previously described but using the forward TTCTGTTGGTGCTGATATTGCAGTAACTGAAAATGCTTCTGAGTGGC-3′ and reverse 5′-ACTTGCCTGTCGCTCTATCTTCTTGAAGAGAGGTGACAAAACAAAGGG-3′ primers. All primer sequences were designed to include 5′ universal overhangs required for posterior barcoding of samples during Oxford Nanopore library preparation (underlined nucleotides). PCR primer specificity was verified using UCSC *in silico* PCR (https://genome.ucsc.edu/cgi-bin/hgPcr). The first PCR produced a 13 kb amplicon while the nested PCR produced a 12.84 kb amplicon.

For DNA library preparation, we used the PCR-amplified *SCN10A* haplotype region from the n = 86 Brugada syndrome cases prepared using the Oxford Nanopore 1D DNA ligation Sequencing Kit SQK-LSK109. The library was prepared following the manufacturer’s recommendations, although we included a few optimizations. All purification steps were performed with Agencourt AMPure XP beads at 0.41X followed with ethanol 75% clean-ups. We also added two extra clean-ups using the Short Read Eliminator XS (Circulomics) to remove unspecific DNA fragments. DNA was end-repaired and dA-tailed using the NEBNext Ultra II End Repair / dA-tailing quick ligation module (New England Biolabs, E7546) in accordance with the Oxford Nanopore protocol. The DNA library was sequenced on a MinION (R9.4 flowcell chemistry) at the Center for Molecular Medicine and Chronic Disease Research (CiMUS, Santiago de Compostela, Spain). Base calling of the raw nanopore reads was performed using the Oxford Nanopore base caller Guppy (v3.2.10) to generate FASTQ files containing sequencing reads files from each Brugada syndrome sample. Reads were aligned to the human reference genome (GRCh37/hg19) using minimap2 (v2.17)^92^ with the *-ont* preset. We used SAMtools to convert minimap2 alignments to BAM format and removed multimapping reads and supplementary alignments. SAMtools was also used to remove reads with soft-clipping and reads smaller than 8 kb (minimum length that we estimated to be required for posterior haplotype phasing).

#### Haplotype and single-SNV association with Brugada syndrome

Haplotypes 1-15 (Hap1-15) in the enhancer block were tested for association with Brugada syndrome using unconditional logistic regression analyses under the assumption of a recessive, dominant and multiplicative models of inheritance. Association results were considered significant when the p value was smaller than 3.33e-03, based on the number of haplotypes tested (0.05/15). In addition to haplotypes, we performed the same disease association analyses for the seven variants forming the haplotypes individually (i.e., rs6801957, rs6799257, rs9836859, rs6790396, rs9874633, rs10428132 and rs10428168; rs1-7, respectively). In the case of single-SNVs, association results were considered significant when the *p-value* was smaller than 7.14e-03, based on the number of SNVs tested (0.05/7). The most likely inheritance model was assigned to each haplotype and single-SNV based on p values and the Bayesian Information Criterion (BIC). Haplotype and single-SNVs logistic regression results are reported as odds ratio (OR) and 95% confidence intervals (95%CI) after adjustment for gender, and they were obtained for all NFE control groups (1KG, Wellderly, and GTEx). All analyses were performed on R v3.5.2.

#### GTEx *cis*-eQTL analysis

A total of n = 202 left ventricle samples with available genotype phasing information were selected to examine the effects of haplotypes and single-SNVs to *SCN5A* and *SCN10A* expression. Gene expression data was downloaded from GTEx through dbGaP under the accession code phs000424.v7.p2^56^ (approved access #82151, May/2020). Genes with less than one read count across all samples were excluded, and expression values for remaining genes were subjected to variance stabilizing transformation (VST) as implemented in DESeq2.^93^ We then used the multi-omics factor analysis (MOFA+)^67^ to account for hidden factors (i.e., technical variation) that represent the driving sources of variation across expression data. We run MOFA –R package version– on default parameters using the VST-normalized read counts as expression input. MOFA identified fifteen factors that were correlated with covariates reported previously for GTEx samples, such as ischemic time. To determine the optimal number of MOFA factors to be regressed out from expression data, we focused on eQTL p values for *SCN5A* and *SCN10A* genes. Briefly, we compared p values obtained by a pairwise t test comparing *SCN5A* and *SCN10A* expression between individuals Hap^1/1^ and Hap^2/3^, after adjusting for different number of MOFA factors.

### Quantification and statistical analysis

Statistical analyses and graph generation were performed in R v3.5.2. Statistical details of the analyses as well as sample numbers (n) are presented in the main text and figure legends. For the case-control association analyses, significance was assessed with Fisher’s exact tests, and pairwise comparisons were corrected for multiple comparisons with the Bonferroni method. For the haplotype versus single-SNV association analyses, unconditional logistic regression analyses were performed under the assumption of a recessive, dominant and multiplicative models of inheritance, after adjusting for gender. Bayesian Information Criterion (BIC) was used to quantify evidence in favor of each inheritance model. For Hap^1/1^ correlation with ECG parameters, a two-tailed t test distribution was performed assuming homoscedastic variance.

## Supplemental information

Data S1 Tables S1–S8

Document S2. Article plus supplemental information
