# Supplementary material for: Analysis of Brugada syndrome loci reveals that fine-mapping clustered GWAS hits enhances the annotation of disease-relevant variants
Source: Cell Rep Med. 2021 Apr 20;2(4):100250. doi: 10.1016/j.xcrm.2021.100250 (PMC8080235; doi:10.1016/j.xcrm.2021.100250)
Supplement: Document S1 — Figures S1–S5 [file mmc1.pdf]

Cell Reports Medicine, Volume *2*

Supplemental information

Analysis of Brugada syndrome loci reveals that fine-mapping clustered GWAS hits enhances the annotation of disease-relevant variants

Mel·lina Pinsach-Abuin, Bernat del Olmo, Adrian Pérez-Agustin, Jesus Mates, Catarina Allegue, Anna Iglesias, Qi Ma, Daria Merkurjev, Sergiy Konovalov, Jing Zhang, Farah Sheikh, Amalio Telenti, Josep Brugada, Ramon Brugada, Melissa Gymrek, Julia di Iulio, Ivan Garcia-Bassets, and Sara Pagans

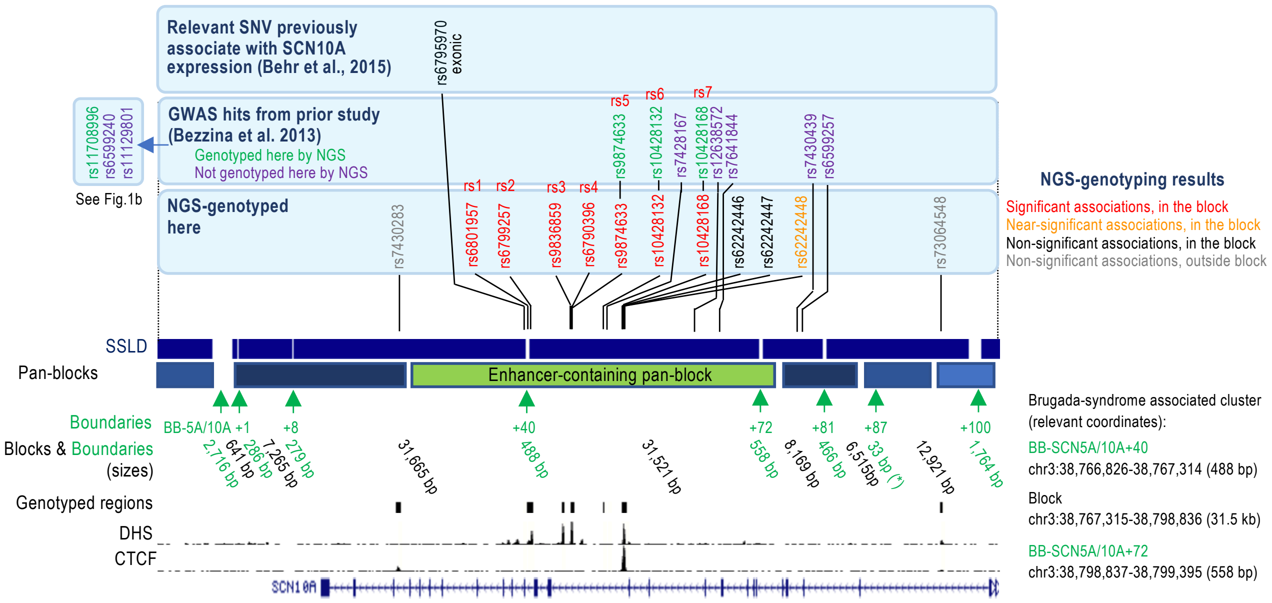

Figure S1. Relevant SNVs at and around the enhancer-containing pan-block (related to Figure 1). Top panel, a coding SNV associated with *SCN10A* reported by Behr and colleagues; middle panel, GWAS hits reported by Bezzina and colleagues; bottom panel, common SNVs genotyped by NGS in this study (those significant associated, in red, will be annotated as rs1-7). Also included a track with SSLD-based block and boundary predictions (* 33 bp boundary at +87 kb; we note that this boundary is too short to be visible in the track of predicted pan-blocks). Color coded SNVs indicated on the side. Block boundaries (BB) and their distance (in kb) to BB-SCN5A/10A indicated; as well as pan-block boundary and pan-block sizes. Region: chr3:38,715,945-38,832,350 (hg19). Other relevant coordinates on the side.

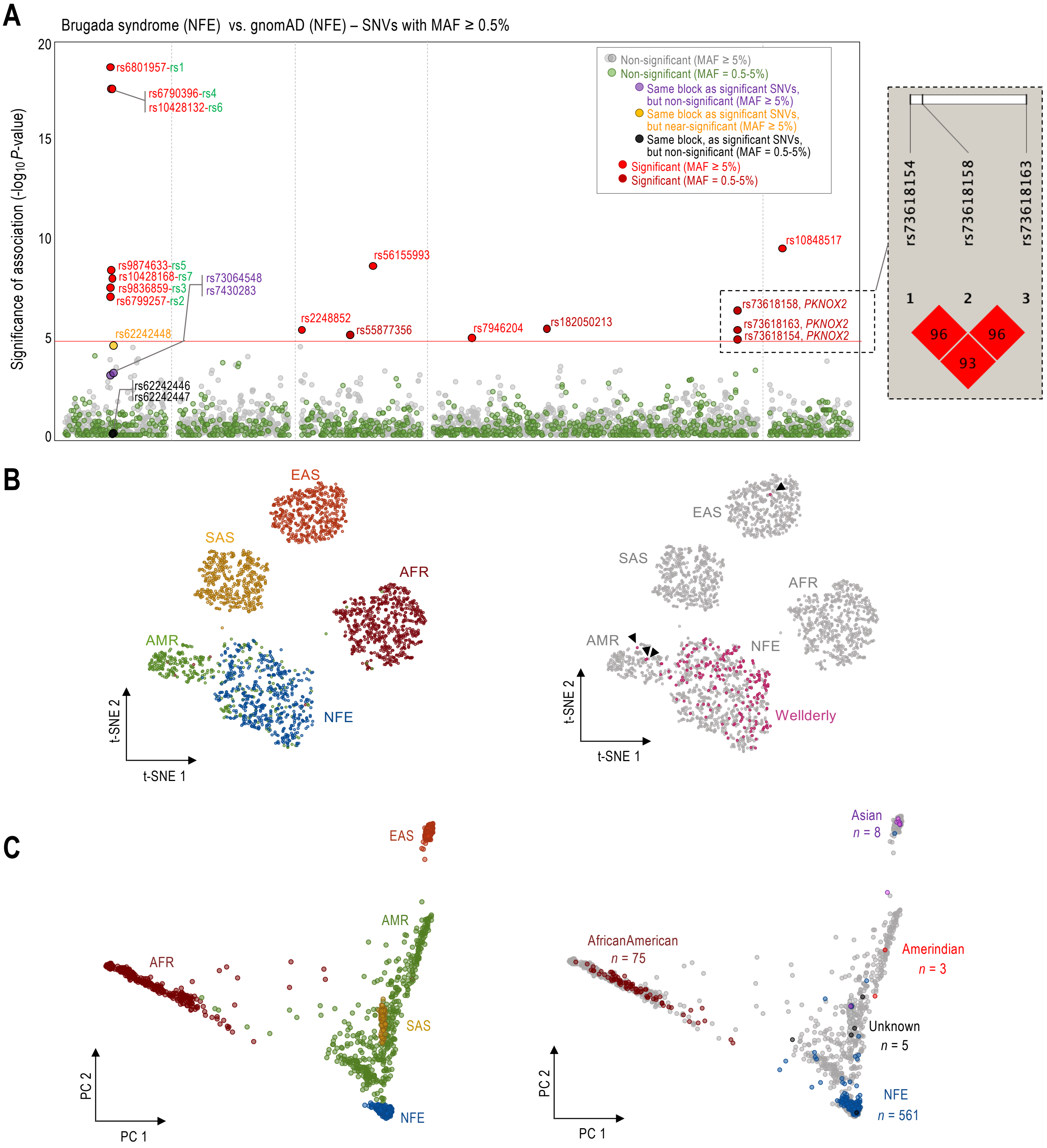

Figure S2. Significance of the associations for low frequency SNVs and ancestry admixture of Wellderly and GTEx datasets (related to Figures 2 and 3). **A**. Manhattan plot shows the significance of the associations for low frequency (MAF = 0.5-5%, in green) SNV alleles investigated in this study and Brugada syndrome in a case-control analysis of $n = 86$ patients using as controls $n = 7,718$ individuals with NFE ancestry in the gnomAD database. Common SNVs, in gray, also included, as in Figure 2D. The red horizontal line marks the threshold of significance, based on Bonferroni corrected α level of $p = 2.35e\text{-}5$ (0.05/2,121) according to the number of common SNVs tested. Labeling of relevant SNVs, as indicated. **B.** t-SNE plot showing the ancestry admixture of $n = 200$ Wellderly individuals using 1KG Phase3 Illumina sequencing data as reference panel. t-SNE plot was performed using the first 6 principal components. *Left panel,* 1KG samples color coded by ethnicity, $n = 2,405$. *Right panel,* samples from 1KG Phase3 shown in gray and Wellderly samples colored. Outlier ($n = 4$) Wellderly individuals not having an NFE descent, highlighted with black arrows, were excluded from further analysis. **C**. Analysis of population structure for $n = 652$ GTEx individuals using 1KG Phase3 Illumina Omni 2.5 genotype array as a reference panel. *Left panel,* 1KG samples color coded by ethnicity, $n = 2,218$. *Right panel,* 1KG samples shown in gray and GTEx samples color coded according to self-reported ancestry (number of individuals, as indicated). Note: from the $n = 561$ GTEx-NFE individuals, $n = 208$ were removed from further analysis for not having phasing information available on GTEx dbGaP (phs000424.v7.p2). Also, $n = 2$ individuals with unknown ancestry were included as they clustered with individuals of NFE ancestry and had phasing information available. Therefore, we included a total of $n = 355$ GTEx-NFE samples).

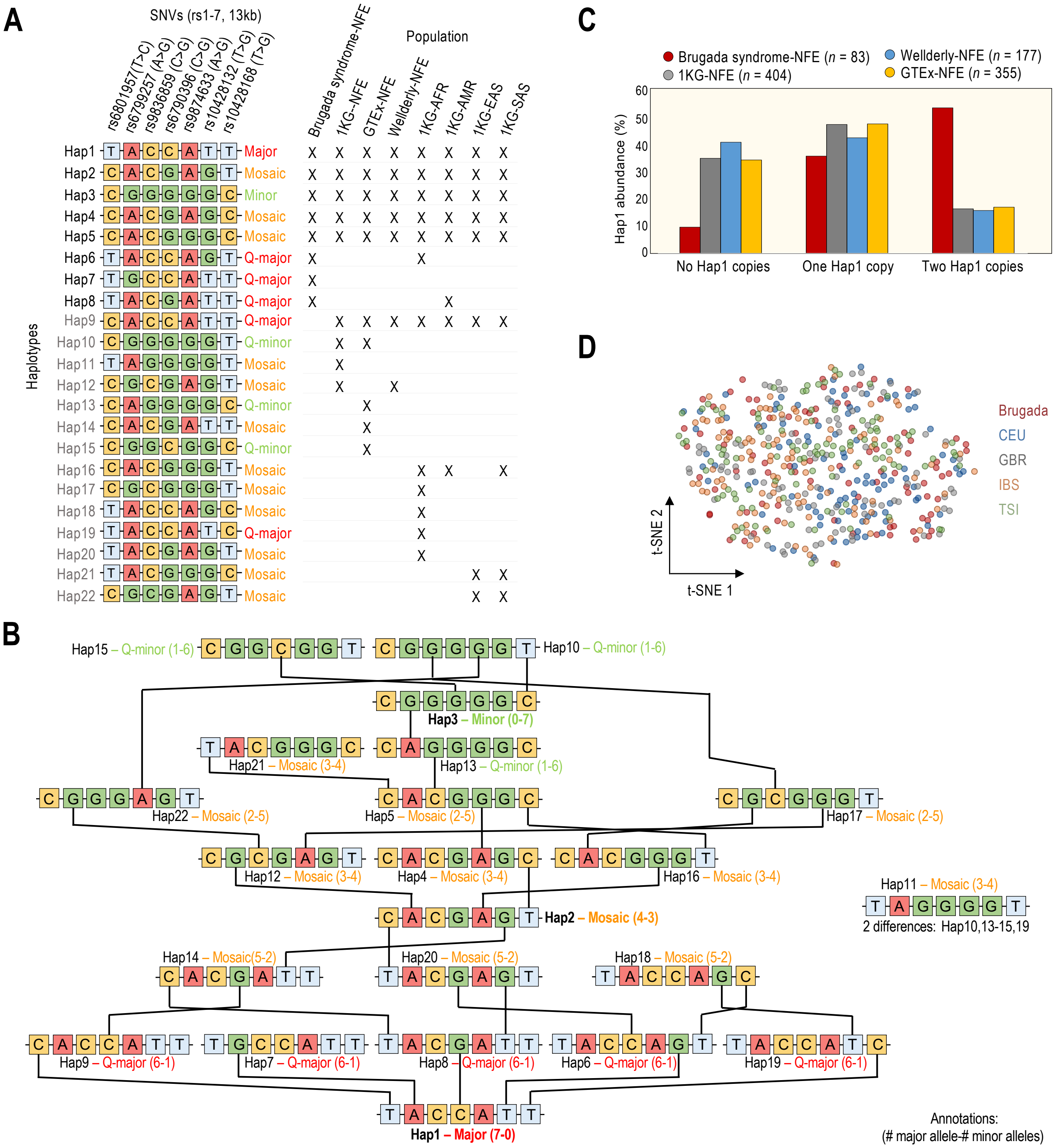

Figure S3. Sequences of haplotypes inferred in this study and abundance of Hap1 in different NFE populations (related to Figure 3). **A.** Haplotypes inferred in this study: Hap1-6/8 (experimentally validated in Brugada patients); Hap7 (inferred in Brugada patients); Hap9-12 (inferred in controls with NFE ancestry, 1KG dataset); Hap13-15 and, 22 (inferred in controls with NFE ancestry, GTEx dataset); and Hap16-22 (inferred in controls with non-NFE ancestry, 1KG database). **B**. Scheme showing Hap1-22 sequences manually organized to indicate allelic differences. Differences in more than one allele are not indicated (e.g., Hap11 shows two allelic differences relative to other haplotypes). The number in parenthesis indicate counts of major (first) and minor (second) alleles. We note that this scheme does not intend to impute phylogeny or to reconstruct a history of mutational events, but it intends to provide a visualization of allelic differences. **C**. Abundance of Hap1 copies among different NFE populations (0, 1, or 2). We suspect that the OR (in Figure 4A) and BIC values (Data S1, Table S6) are significant for the multiplicative model because the proportion of Brugada individuals presenting Hap1 genotype increases with the number of Hap1 copies. However, carrying one Hap1 copy (Hap$^{1/-}$) is less frequent in Brugada than in the 3 control NFE populations analyzed (1KG, Wdy and GTEx). For this reason, we favor the recessive model over the multiplicative. The multiplicative model, we think, is artificially enhanced because the number of individuals carrying no Hap1 copies is highly underrepresented in the Brugada cohort, primarily because there is a protective genotype in the population, Hap$^{2/3}$. **D**. t-SNE plot showing the ancestry admixture of *n* = 86 Brugada individuals using *n* = 404 NFE individuals from 1KG Phase3 Illumina sequencing data as reference panel. 1KG samples color coded by sub-population; *n* = 99 CEU, *n* = 91 GBR, *n* = 107 IBS, *and n* = 107 TSI. As expected, we do not observe segregation among NFE subpopulations due to the resolution of our analysis that is limited to the *n* = 1,293 genomic regions sequenced (or ~1 Mb of the genome).

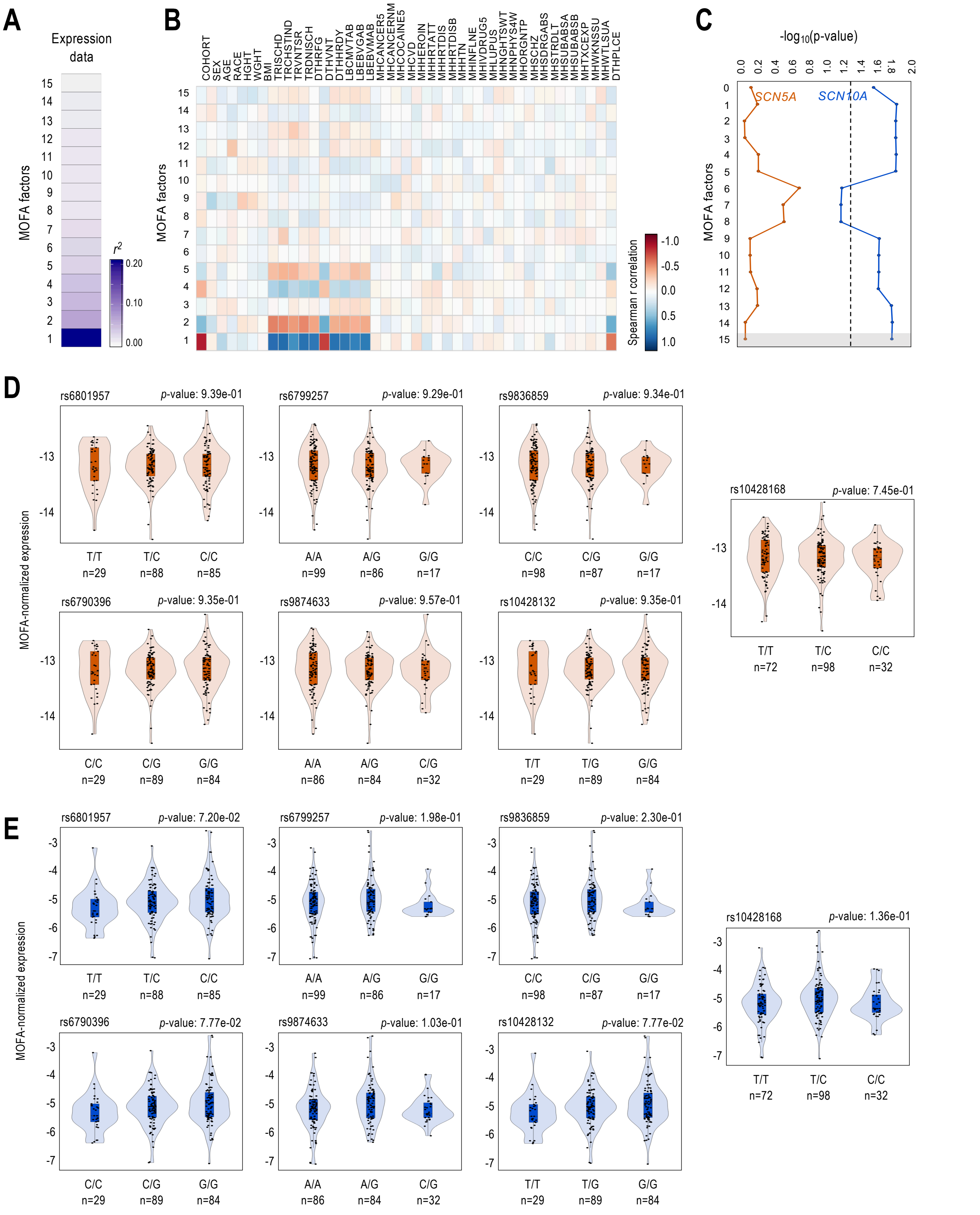

Figure S4. Application of MOFA to left ventricle samples and *cis*-eQTL analysis of individual rs1-7 on *SCN5A* and *SCN10A* (related to Figure 4)**. A.** Application of MOFA to n = 202 left ventricle samples. Proportion of total variance explained (r^2) by individual factors using left ventricle GTEx expression data. **B.** Spearman correlation coefficients of MOFA factors (y-axis) versus data processing covariates (x-axis)–as defined for the GTEx cohort in dbGaP study phs000424.v7.p2. Color scale ranges from blue (r = 1) to white (r = 0) to red (r = -1) of Spearman correlation. **C.** Effect of varying number of MOFA factors on eQTL p-values ($-\log_{10}$) obtained when comparing *SCN5A* and *SCN10A* expression distribution for individuals with $Hap^{1/1}$ versus individuals $Hap^{2/3}$ (see Methods). Dashed horizontal line represents the p-value significance threshold of 0.05. Gray shadow highlights the final number of MOFA factors to be regressed out. **D-E.** *cis*-eQTL analysis of individual rs1-7 on *SCN5A* (D) and *SCN10A* (E), using expression data of human left ventricle tissue generated by GTEx (no ancestry selection; MOFA-corrected expression). Violin plot shows median expression and box indicating interquartile range and sample point (number also indicated). Significance tested by one-way ANOVA and Tukey Honest Significant Differences (Tukey HSD) test for multiple pairwise-comparisons. Significance threshold, $p < 0.05$.

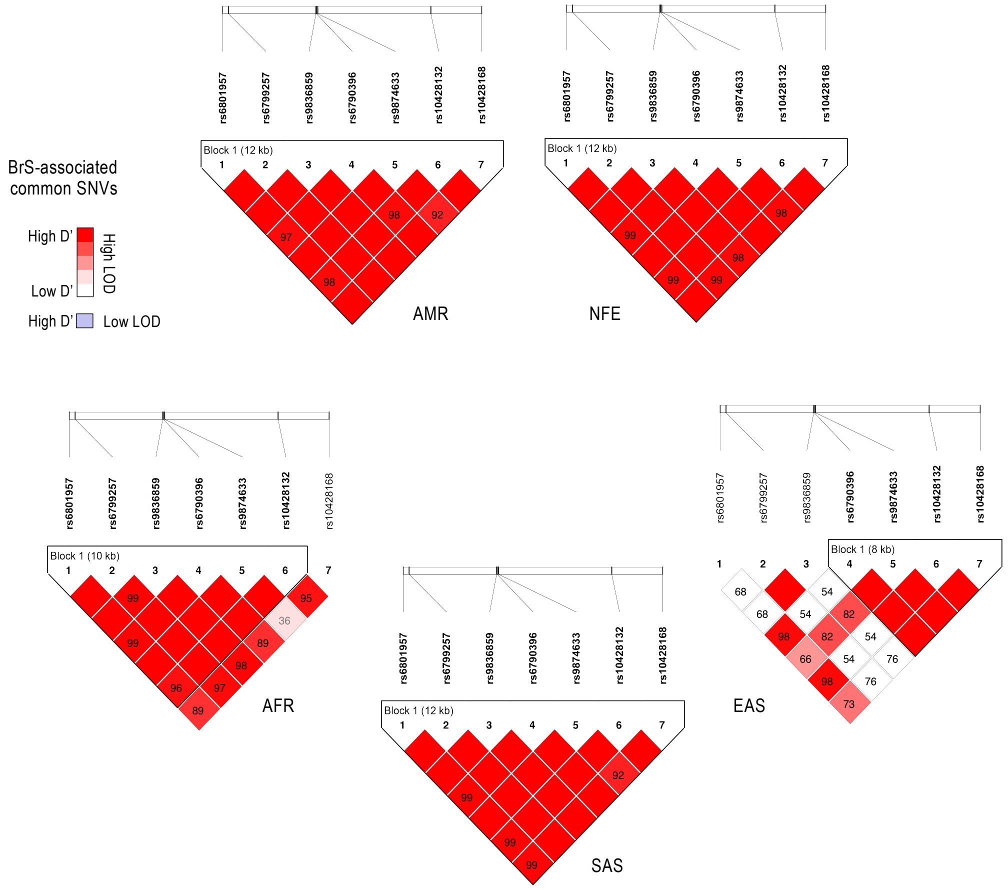

Figure S5. LD for the seven SNVs associated with Brugada syndrome downstream the inferred BB-SCN5A/10A+29 site (related to Figure 5). These SNVs are in strong LD in only some human super-populations of the 1KG project dataset. Heatmaps generated with Haploview. Color scheme based on 100x D' values (values indicated unless D' = 100), and log of the likelihood odds (LOD) ratios.
